# Practice-dependent changes in body representation but not in reaching distance following virtual tool-use

**DOI:** 10.1371/journal.pone.0361297

**Published:** 2026-10-27

**Authors:** Amir Jahanian Najafabadi, Christoph Kayser

**Affiliations:** Department of Cognitive Neuroscience, Bielefeld University, Bielefeld, Germany; University of Hamburg, GERMANY

## Abstract

Previous research has shown that tool-use can modify neural representations of our body and the space surrounding us. Furthermore, virtual tools can become incorporated into the user’s body representation after training. However, the extent to which such changes depend on the amount of sensorimotor experience remains unclear. In the present study, we investigated whether different amounts of virtual tool-use training (30, 60, 90 and 120 trials) influence body and action-oriented spatial representations. Participants completed a tactile distance judgment (TDJ) task, indexing body representation; and a reaching distance estimation (RDE) task, indexing perceived actionable space, before and after virtual tool-use training. Virtual tool-use produced an overall decrease in TDJ responses, indicating plastic changes in the forearm tactile distance perception. Although the magnitude of change varied across training conditions, the strongest and most reliable evidence for TDJ change emerged following 120 trials of practice, suggesting that extended sensorimotor experience may be required for stable modifications of body representations. In contrast, RDE changed modestly from pre-test to post-test but did not vary systematically with training duration, suggesting that perceived reaching distance may adapt more rapidly and with less dependence on additional practice. Arm length was a significant predictor of RDE error, indicating that stable anatomical constraints continue to influence reachability judgments despite tool-use experience. Together, these findings suggest a dissociation between body representation and perceived reaching distance, with each exhibiting distinct temporal dynamics following tool-use training. More broadly, the results demonstrate that virtual tool-use can induce changes that transfer to subsequent physical assessment, highlighting virtual environments as a valuable framework for investigating body representation plasticity and tool embodiment.

## 1. Introduction

Interacting with our surrounding using tools can have profound influences on how the brain represents both our body and the space surrounding us [1–7]. To this end, previous work distinguishes between the representations of our body itself and that of peripersonal space. Body representation refers to a somatosensory-driven internal model of the body's metric properties (e.g., limb length and shape) that informs movement planning, motor control and bodily perception [8,9]. In contrast, peripersonal space is defined as the space surrounding the body within which objects can be reached and acted upon [10–12]. Recent studies show that both the perceived, or implicitly assumed, size of the involved body parts and that of our peripersonal space can adapt following tool-use. In fact, while tool-use can expand the size of our reachable space, it can lead also to the morphological updating of the implicit body representation and the perceived size of the involved limbs [6,13–23].

Within the broader framework of body representation, the dyadic model distinguishes two principal subcomponents: the body schema and the body image. The body schema is typically described as an unconscious sensorimotor representation that supports movement planning, online motor control, and the integration of sensory feedback during action. In contrast, the body image refers to a more conscious and perceptual representation of the body's size, shape, and metric properties [24,25]. Both representations contribute to action and bodily perception and are continuously updated through sensorimotor experience and interactions with the surrounding environment [6,13,14,19,26–28]. This distinction is particularly relevant for tool-use research because tool-use may influence body, and space-related representations through partly different mechanisms. Following the conceptual criteria proposed by de Vignemont, the body schema can be investigated using tactile distance judgment (TDJ) tasks, which assess how tactile distances are mapped onto an internal representation of the body surface [29]. In contrast, when individuals estimate whether an object is reachable, judgments are influenced by their representation of bodily dimensions, such as arm length, as well as their perceived action capabilities. Consequently, reaching distance estimation (RDE) is best interpreted as a behavioural measure of perceived reachability or reachable action space rather than as a direct measure of body schema, body image, or peripersonal space. Nevertheless, body-related representations remain important for such judgments because estimates of reachability depend on the perceived dimensions and capabilities of the body [27].

Importantly, peripersonal space should not be equated with reachability space. Although the two constructs overlap, they refer to partly distinct spatial representations. Peripersonal space is typically body-part centred and closely linked to multisensory processing near the body, whereas reachability space reflects the broader region within which actions can be performed based on current action capabilities, such as arm length, posture, and tool-use possibilities [30]. Consequently, an object may be reachable without necessarily lying within the immediate multisensory space surrounding a specific body part. Accordingly, the RDE task should be interpreted as a behavioural measure of perceived reachability or reachable action space rather than as a direct measure of peripersonal space boundaries.

### 1.1. Tasks to measure body and spatial representations

To examine changes in the somatosensory-driven model of the body, previous work has relied on TDJ [13,17,20,21,31–34]. TDJ measures how individuals estimate the distance between two tactile stimuli delivered on a body part, such as the forearm. Comparing TDJ performance before and after tool-use allows measuring tool-induced modifications of the body representation. This has been central to demonstrating that perceived tactile distances are recalibrated to account for tool incorporation. Tool-use induced changes in TDJ are thought to reflect a reorganization of neural representations of the body, where the tool's functional length is integrated into the limb's metric representation [4,15,17,20,31,35]. As a result, changes in TDJ accuracy can be considered a meaningful indicator of representational plasticity arising from tool-use [17].

Perception of tactile distance depends on an implicit representation of body morphology rather than solely on the physical properties of the tactile stimulus [4]. This view is consistent with the proposal that perceived body-part size (body image), proprioceptive information, and the representation of the body surface (body schema) are coherently organized within body representation [31]. Thus, tactile perception is shaped not only by exteroceptive input from the skin, but also by internal representations specifying the size, shape, and spatial layout of the body. Therefore, if virtual tool use induces plastic changes in body representation, corresponding changes should be reflected in tactile distance perception, that is, in the perceived size of objects contacting the skin surface [20]. In the present study, we assessed TDJ on the forearm before and after virtual tool-use training to determine whether sensorimotor experience leads to a change in the internal representation of the body. Furthermore, by manipulating the amount of tool-use practice (30, 60, 90, and 120 trials), we examined whether the magnitude of body representation plasticity scales with the extent of sensorimotor experience.

To examine changes in the action-oriented representation of the reachable space, previous work measured RDE. RDE relies on the integration of bodily metrics, body schema, body image, visual information, and action-related spatial computations to estimate whether and how far an object can be reached [26,27,29,36] and it assesses perceived reachability both within and beyond the immediate peripersonal space. However, it should not be treated as a direct or exclusive measure of peripersonal space boundaries. Rather, RDE provides a behavioral index of how bodily metrics are integrated with action-related spatial judgments. Consequently, changes in RDE reflect adjustments in the perceived limits of reachable action space rather than structural changes in body representation per se [6,15]. Unlike TDJ which is used to measure implicit representational plasticity and primarily targets limb-centered metric perception based on tactile information, RDE relies on a combination multisensory signal, including proprioceptive, visual, and motor information [5, 12]. In previous work [27], we used the RDE task to assess whether and how participants’ perception of reachable distance changes as a result of the tool-use experience relative to their arm length. Because effective tool-use requires the brain to treat the tool tip as the functional boundary for action, shifts in RDE are interpreted as recalibration of perceived action space relative to physical arm length [37,38].

Conceptually, TDJ and RDE provide complementary measures of body and space around it at different spatial scales. TDJ reflects local metric properties of the measured limb by indexing how distances on the skin are internally represented. In contrast, RDE reflects a more global, action-oriented representation of the body in external space, requiring the integration of arm length, movement capabilities, and perceived action boundaries. Tool-use may induce plasticity at both levels, such that changes in limb representation are expressed locally in tactile distance perception and more globally in perceived reachable space. Importantly, in a recent study we found that these measures do not necessarily need to covary [27,36], and dissociations between TDJ and RDE can offer insight into the specificity of tool-induced plasticity across different components of body representation.

### 1.2. The present study

Previous studies on the impact of tool-use on body and spatial representations are highly diverse, leading to discrepancies in both the presence and the magnitude of reported effects [13–15,17,19,20,27,39]. Regarding participants age, research has shown that the susceptibility to tool-induced changes is not uniform across the lifespan; while younger adults often show robust plasticity in both TDJ [14,15,20], older adults may exhibit a diminished or even absent response in somatosensory metrics despite showing changes in action-oriented space measured through RDE [27,36]. This suggests that age modulates the degree of representational plasticity, with certain components of the self-system becoming more resistant to change with experience. Furthermore, both the mode of training – physical versus virtual -, and the practice dose required to produce stable effects remain highly inconsistent across studies [17,33,40]. In particular, the amount of training required to induce stable effects proves very inconsistent across studies. While some used 15–30 trials [41–44], others used 48 trials [18,19], 60 trials [27], or longer sessions involving 120–240 trials [17,32,45]. Others have used duration-based protocols with unrestricted trial counts, ranging from 8 to 16 minutes [20], 20 minutes [46], or 16 minutes [38]. For example, Cardinali et al. reported that just 10 minutes of training with a mechanical tool extending the arm altered the perceived morphology of the limb [14]. This led participants to perceive their arm as longer and subsequently altered the kinematics of their reaching movements as if they were using a longer limb. However, a recent tool-use study across the life span showed that 60 trials training with either a small or larger tool length used to grasp objects at near or far distances from the body did not lead to changes in forearm representation in any age group [27]. This suggests that the relationship between tool-use and body representation is not a simple binary “on/off” phenomenon. Instead, it appears to be modulated by factors that have yet to be systematically isolated, most notably the duration of practice. While some models propose that tool integration can occur almost immediately following successful goal attainment [47], the failure of 60-trial protocols to replicate these effects points to a more complex, potentially non-linear, dose-response relationship.

Also, the nature of tool-use training varies considerably across studies, particularly with respect to the sensory-motor demands imposed by the tool itself. Changes in perceived tactile distance on the forearm have been reported across a range of interfaces, from simple reach-extending mechanical pointers [20] to more complex functional tools such as rakes, which require integration of distal “hooking” or “grabbing” dynamics [15]. Similar effects have also been observed with virtual tools in virtual reality [17], where physical tactile feedback is replaced by visual or vibrotactile substitutes or possibly even absent. Across these tasks, participants may manipulate objects at a distance or pull them toward the body, suggesting that the direction and goal structure of the action may be as important as the physical properties of the tool. Accordingly, it remains unclear to what extent specific training constraints, such as the degree of physical resistance or the complexity of the tool’s functional end, determine the magnitude of changes in body and spatial representations. For instance, if training effects transfer from a virtual environment to physical tactile and reaching distance judgments, this would provide strong evidence that tool-use updates higher-order, amodal representations of body and space rather than only representations directly tied to a representation anchored in a very specific form of sensory signals [48]. Hence, such a transfer would imply that the brain forms a generalized internal model of the tool-plus-limb system that is not tied to the particular sensory context, virtual or physical, in which the action was acquired.

Resolving these questions concerning the amount of training necessary to induce changes in body representations and the transfer between virtual and physical environments was the primary motivation for the current study. By systematically manipulating the number of training trials, we aim to determine the specific amount of practice required to induce stable changes in both forearm tactile distance perception (measured by TDJ) and reaching distance estimation measured by RDE task [26,27,33,36]. At the same time, we relied on a tool-use training paradigm implemented in virtual reality, following a testing tool-induced plasticity in the real-world, i.e., on participants’ actual arm. This allowed us to test how virtual training translates to the actual physical body and its surrounding. Participants performed a virtual reality-based tool-use task that involved grasping and manipulating objects. The training was divided into four blocks – 30, 60, 90, and 120 trials presented in counterbalanced order over the course of two days. Practically we quantified the following based on our experimental data: **First,** how participants estimate tactile distances on the forearm as well as reaching distance at baseline, and whether they tend to over- or underestimate these distances prior to any tool-use training. **Second,** by how much perceived forearm tactile distance changes following virtual tool-use, and how this is influences by the amount of practice. **Third,** how the shift in perceived reaching distance after tool-use training depends on the amount of practice. Based on the functional differences between body representation and reachable action space, we hypothesized that virtual tool-use training would induce a practice-dependent changes in TDJ administered in the right forearm, with the most stable and pronounced changes emerging only after extended sensorimotor exposure (120 trials). Conversely, we hypothesized that RDE would show a rapid, practice-independent adjustment, reaching a functional asymptote immediately following early successful goal attainment (30 trials) irrespective of the further training duration.

## 2. Method

In total, thirty-four volunteers aged between 18 and 35 years old were recruited. Two participants were excluded for further analyses because of incomplete datasets, resulting in a sample of thirty-two participants. Sample size was estimated based on the tool-use effect reported by Miller et al. [20], particularly the arm-shaped-tool/arm condition, where tactile distance perception changed significantly following tool-use. Reconstructing the paired-samples effect size from the reported statistics yielded a conservative effect size of approximately Cohen’s d = 0.86. A two-tailed paired-samples power analysis with α = .05 indicated that 13, 17, and 20 participants would be required to achieve 80%, 90%, and 95% power, respectively. To allow for exclusions and to provide stable estimates across training-dose conditions, we therefore aimed to recruit at least 30–35 participants. The data were collected anonymously and prior to participation, digital informed consent was obtained from all individuals. Each participant had either normal vision or vision corrected with glasses, no history of neurological disorders, and was right-handed. To prevent potential biases participants were not briefed on the study's specific hypothesis. Compensation of 13 Euros per hour was provided. The study was conducted between 01/04/2024 until 31/08/2024. This study was approved by the Ethics Committee of Bielefeld University (EUB-2023–120-S) according to the ethical guidelines of the German Association of Psychology (Deutsche Gesellschaft für Psychologie; DGP), which correspond to the guidelines of the American Psychological Association (APA).

### 2.1. Experiment design

The overall design was as follows (cf., Fig 1). Participants performed tool-use training in a virtual environment and learned to control a virtual mechanical grabber performed on different days. In each day, two blocks comprising a total of 150 training trials (either 30 and 120, *or* 90 and 60) were performed in a virtual environment. Subsequently to each block forearm tactile distance perception was assessed with a well-established TDJ task (for details see section 2.3.1) where participants judged distances between two tactile stimuli applied to their right forearm. Perceived reaching distance was assessed by a RDE tasks adapted from prior research in relation to participants actual arm length (for details see section 2.3.2; [27,33,36]. TDJ and RDE were measured before and after each training block.

**Fig 1:**
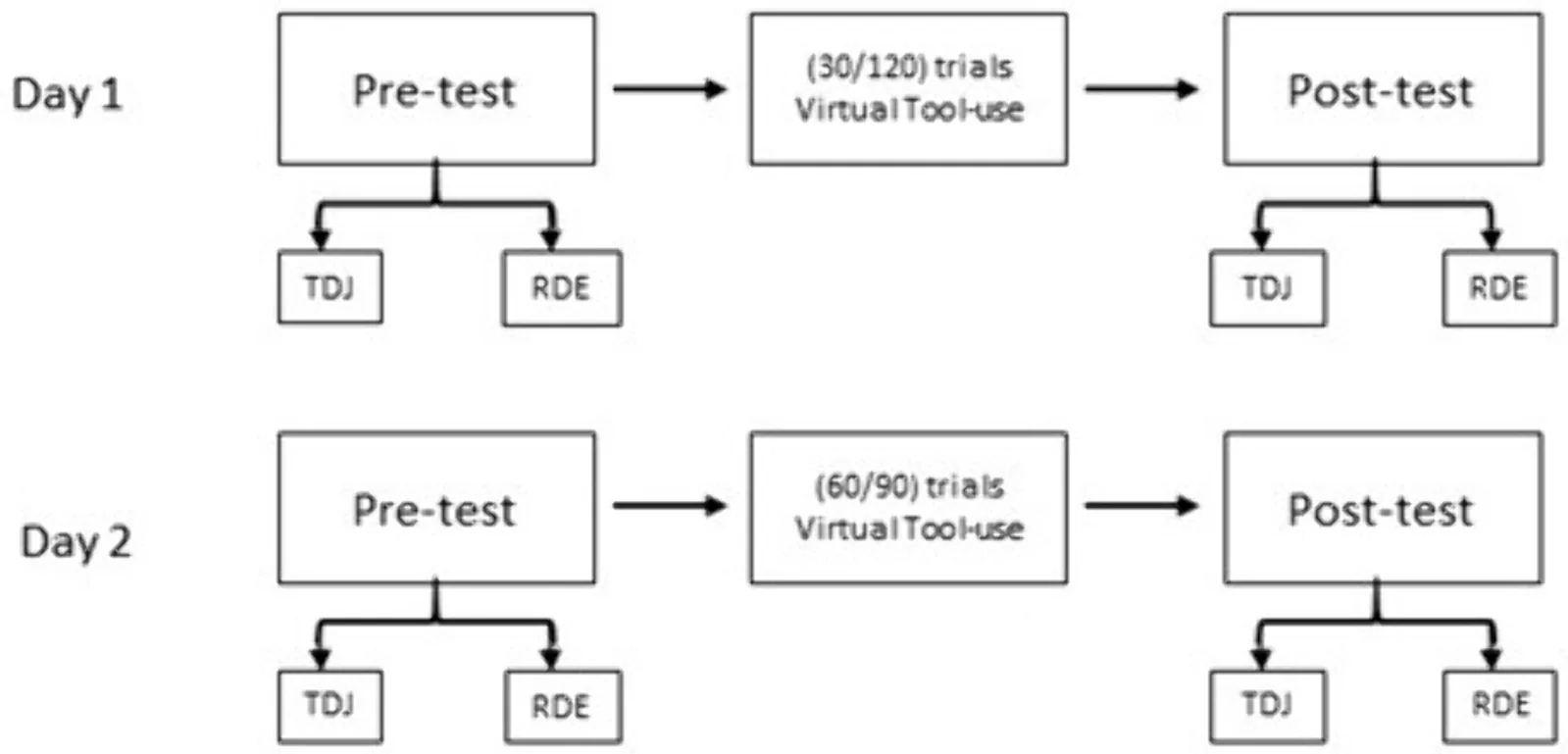
Experimental design. On each day, participants completed a pre-test followed by a block of virtual tool-use training to learn control of a virtual mechanical grabber and then a post-test. Pre- and post-tests included a tactile distance judgment (TDJ) task assessing forearm tactile distance perception and a reaching distance estimation (RDE) task assessing perceived reaching distance. Each day comprised two training blocks (30 and 120 trials on Day 1; 60 and 90 trials on Day 2; total of 150 trials per day), separated by a 30-minute rest period to minimize carry-over effects. The order of training blocks within each day was counterbalanced across participants.

To isolate the effects of training duration and minimize carry-over between blocks, a 30-minute rest period was imposed between the two practice blocks on the same day, in line with recent tool-use research [17,27,32,33,36]. This washout interval was selected based on previous evidence indicating that tool-use–induced modulations of somatosensory cortical responses can persist for approximately 10 minutes following training [35]. In addition, the order of the two tool-use training blocks (e.g., 30 and 120) within each testing day was counterbalanced across participants to control for potential order effects. On Day 1, the blocks consisted of 30 and 120 trials; on Day 2, of 60 and 90 training trials.

To further reduce carry-over effects between testing days, the two days were separated by one week (±1 day). Previous research has shown that somatosensory perceptual changes induced by brief periods of motor learning can remain detectable for at least 24 hours [49]. Although the persistence of virtual tool-use effects was not directly examined in the present study, this evidence suggests that training-induced sensorimotor adaptations may extend beyond the immediate post-training interval. The one-week separation was therefore implemented to minimize residual effects of prior training and increase the independence of the two testing days. Taken together, the washout interval, within each testing day counterbalancing (between two-blocks), and multi-day separation were implemented to minimize contamination between training conditions and ensure that observed changes could be attributed primarily to the experimental manipulations rather than residual effects of previous tool-use experience.

### 2.2. Virtual tool-use training

Training was performed in a virtual environment (Fig 2). A virtual grabber-like tool was developed in Unity (version 2023.1.7) to train participants at a distance of approximately 120 cm from their body (see Fig 2). The training took place in virtual reality using an HTC Vive Pro 2 headset and HTC Vive Pro Controller (Model 2PR7200) with SteamVR Tracking. The experimental procedure followed those of previous studies [17,32,45]. The virtual tool was modeled to closely resemble a real-world grabber, approximately 75 cm in length, with two hinged claws attached to a long stick and a button to activate the grip. Hinge joints were used to simulate the mechanical behavior of the claws, so that pressing the trigger on the Vive controller caused the virtual gripper to close gradually, replicating the function of a physical tool. During training, participants interacted with virtual 3D cubes, which they were instructed to grasp and move toward their body, placing them into a designated area on a virtual table. The virtual reality table was spatially aligned with a physical table, allowing participants to feel its surface when using either their hand or the virtual tool. The virtual tool was visually anchored to the participant’s right hand, providing a realistic interaction experience.

**Fig 2:**
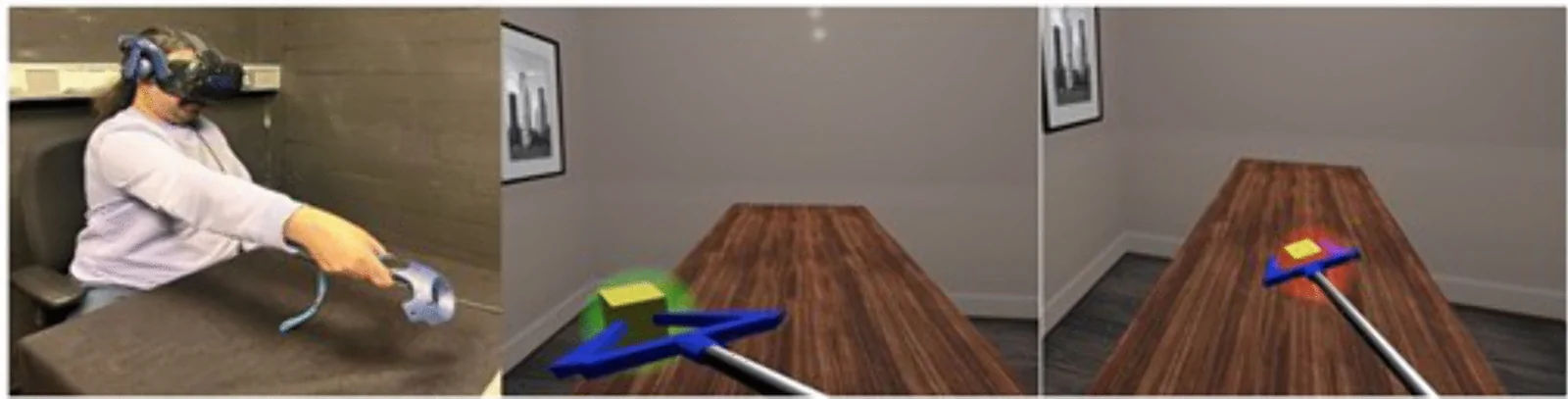
Virtual tool-use training in immersive virtual reality. Left: Participant controlling a virtual grabber while seated at a physical table using an HTC Vive Pro 2 headset and Vive controller (SteamVR tracking). Middle and right: First-person views of the virtual environment showing the grabber interacting with a target cube on a virtual table that is spatially aligned with the physical table surface. During training, participants grasped and moved cubes from a start location to a designated target area at an approximate distance of 120 cm using a grabber-like tool (approximately 75 cm). Task difficulty was adaptively titrated by reducing the gripper aperture in 2% steps using a 3-down/1-up staircase procedure, with visual feedback indicating successful (green) and unsuccessful (red) trials. The individual in this manuscript has given written informed consent to publish these case details.

Each training day included two blocks of virtual tool-use trials, presented with visual feedback. A trial began when a new cube appeared and ended either when the participant successfully grasped and moved the cube into the target area within 15 seconds, or when the time limit was exceeded. Successful completion of a trial was confirmed by visual feedback (green light), triggered when the object was correctly enclosed by the gripper’s tip. The sides of the target cube were 80 × 80 mm^2^. Each training block of a specified number of trials began with the gripper hinges maximally open at 120% of the cube width (i.e., 80 mm^3^). The gripper aperture was adjusted adaptively across trials by reducing its width in 2% steps according to a 3-down/1-up staircase procedure. After three consecutive successful grasps the aperture was decreased, whereas a single failed grasp triggered an increase, thereby titrating task difficulty based on performance while providing trial-by-trial feedback. This staircase converges on an accuracy level of approximately 79.4% correct across practice trials [50]. Errors were indicated by red visual feedback and occurred under the following conditions: holding or pushing the cube for more than 2 seconds, moving it outside the play area (e.g., off the table), or contacting the cube with the wrong part of the gripper (e.g., the sides of the claws). In such cases, the trial was marked as failed and automatically ended. Participants returned their hand to the starting position to begin the next trial.

This adaptive procedure was used to equate task difficulty across participants, maintain engagement, and prevent ceiling or floor effects, thereby ensuring that performance remained in a sensitive range for learning-related changes and embodiment measures.

### 2.3. Measurements

#### 2.3.1. Tactile distance judgment (TDJ) task.

The stimuli for the TDJ task were administered to the participants’ right forearm to investigate changes in the tactile distance perception of the hand using the tool. The TDJ task was completed as pre-test and post-test after each training block (adapted from prior research [17,27,32,33]. During this task, participants comfortably sat on a chair and placed their right hand on the table. The stimuli were presented using four wooden blocks on which 4 screws were mounted, each with different distances between them. The screws had round tips with 9 mm diameter. For TDJ testing parallel to the arm axis (“proximodistal” alignment), 4 sample pairs with distances of 20, 40, 60, and 80 mm were administered randomly (cf., Fig 3, left). Each trial lasted approximately 1 s. Each sample block was presented 5 times, resulting in 20 trials in total (5 trials each per 4 distances).

**Fig 3:**
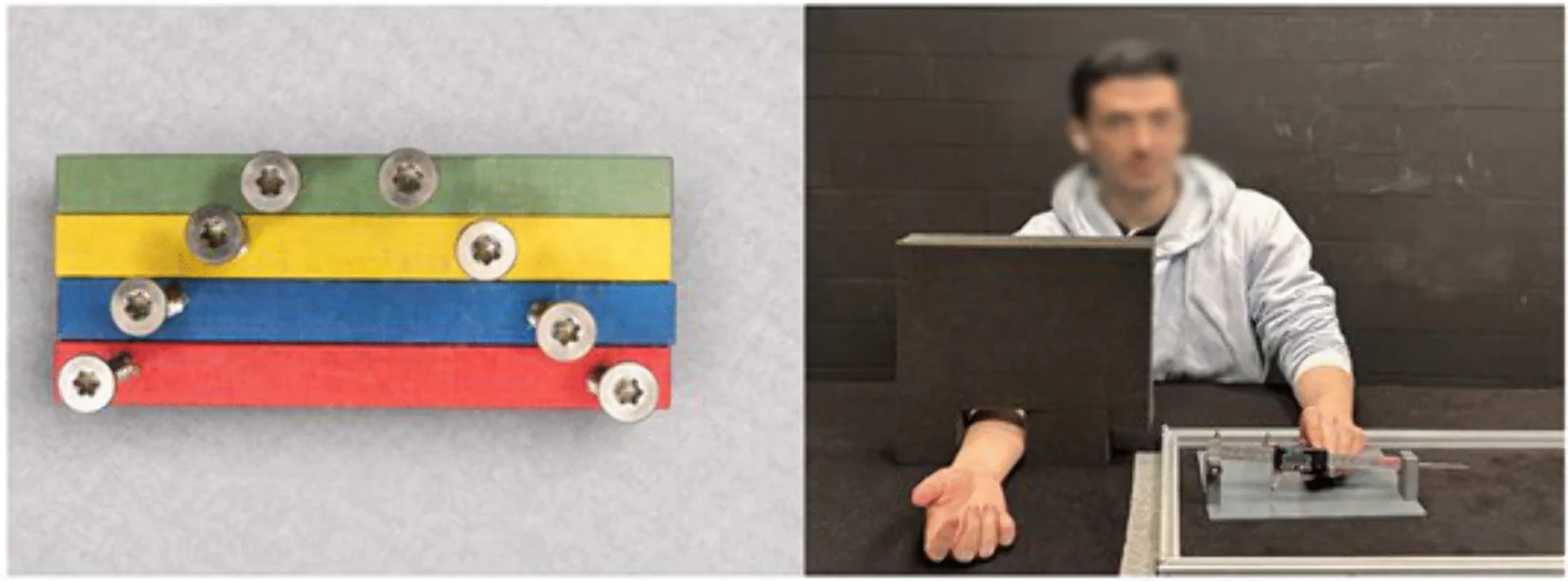
Tactile distance judgment (TDJ) task. *Left:* Wooden stimulus blocks containing pairs of rounded screw tips (9 mm diameter) separated by 20, 40, 60, or 80 mm for proximodistal stimulation on the right forearm. *Right:* Experimental setup showing the participant’s right forearm occluded from view by an opaque separator while distance estimates are reported using a caliper positioned near the left hand. The individual in this manuscript has given written informed consent to publish these case details.

Immediately after stimulus presentation, participants were asked to report absolute distance estimates [17,32,33,36]. For this, two steel legs were fixed to a piece of wood, which in turn was attached to the table close to the participant’s left hand to use a caliper. The participants were able to adjust the distance between the legs with ease. Using their left hand, participants reported the perceived distance by adjusting the distance between the caliper legs. This was displayed on the caliper monitor through an mm scale and was recorded by the experimenter accordingly. The stimuli presented to the forearms of the participants was invisible to the participant due to an opaque separator being placed between their eyes and the right forearm throughout the TDJ test. The goal of this measure was to ensure no visual information would be obtained about the real distances, and whether the pairs of stimuli were administered on proximodistal orientation (cf., Fig 3). We measured how participants judged the distance between pairs of stimuli by looking at the differences (judgment error = estimated distance – actual distance). A positive value indicates an overestimated distance; a negative and underestimated distance. These errors were averaged across five attempts at each distance, and calculated separately for the pre-test and post-test. If participants’ distance judgments decreased after using the tool, it would suggest that the tool had been integrated into how their brain represents their body, specifically within their existing body schema. As a result, their arm would feel shorter than before, and points along the forearm would seem closer to each other than they did initially.

To determine whether changes in TDJ performance reflected genuine modifications of forearm tactile distance perception measured by TDJ rather than nonspecific response biases, a complementary psychophysical regression analysis was performed following prior research [51]. For each participant and separately for each of Pre-test and Post-test, estimated tactile distance was regressed onto actual stimulus distance (20, 40, 60, and 80 mm) according to the following linear model:


$$\begin{array}{c} EstimatedDistance=\beta_{0i}+\beta_{1i}(ActualDistance)+\epsilon_{i} \end{array}$$

where *β₀ᵢ* represents the participant-specific intercept, reflecting a systematic response bias independent of stimulus distance, *β_1_ᵢ* represents the participant-specific slope, reflecting perceptual gain and the degree to which perceived distance scales with actual stimulus distance, and **εᵢ** denotes the residual error. The resulting intercept and slope estimates were then compared between the Pre-test and Post-test sessions using paired-samples *t*-tests. Within this framework, changes in the intercept indicate shifts in general response tendencies, whereas changes in the slope indicate changes in the mapping between actual and perceived forearm tactile distance.

#### 2.3.2. Reaching distance estimation task (RDE).

In this task peripersonal space is operationally defined by estimated reachability along the arms’ length similar to recent research [27,33,36], and tested using procedures adapted from D’Angelo et al. [26,27,37,52]. The RDE task used a small ball (40 mm in diameter), controlled by the experimenter through a linear actuator, that moves towards or away from the participants. The ball's starting position was 33 cm from the participants’ sternum in the withdrawing trials, and at 133 cm in the approaching trials. Participants were asked to estimate the reaching distance, i.e., when they would be able to reach the ball for the last/first time in the withdrawal/approach condition, respectively. These opposing directions allowed us to assess how the initial object position influences reachability judgments [53,54]. During the task participants were instructed to keep their hands loose behind their bodies. Participants could fine-tune the ball distance by asking the experimenter to move the ball slightly further or forward. Finally, between trials, they were asked to close their eyes. The task was repeated for 6 trials, counterbalancing approaching and withdrawing trials. Participants were precisely instructed as “I will now move this target towards you. Please say stop if you think you could touch it with your arm outstretched”. The distance of the ball to the tip of the linear actuator in the final position was measured and recorded for both the approach trials (three trials) and the withdrawal trials (three trials). This score was added to 33 cm and considered as their reported reaching distance. To neutralize the effect of the arm’s length, as a confounding variable, we subtracted the arm’s length from the RDE’s response, following procedures similar to those used by Bourgeois et al. [37]. Participants’ measured arm length was subtracted from the mean scores of the six trials, resulting in an overall distance estimation error in cm. Positive scores, i.e., overestimation of the reaching distance, indicate a perceived extension of the peripersonal space beyond the own arm, while negative scores suggest perceived underestimation of the peripersonal space and arm reaching distance.

## 3. Data preparation and analysis

Data analysis was performed using MATLAB R2022B and figures were produced using Python version 3.13 and was based on the data from thirty-two participants. The primary data included TDJ error and RDE error, calculated as perceived minus actual distance, with negative values indicating underestimation. For TDJ responses, outliers were identified within each participant and task as values exceeding ±2 standard deviations from that participant’s mean estimation error and were excluded from further analysis (removed TDJ outliers = 182 trials in total ≈ 3.6%). No outliers were identified for RDE responses.

Data analyses were conducted using linear mixed-effects models to account for the repeated-measures structure of the data. For TDJ and RDE separately, Session (Pre-test vs. Post-test) and Trial Block (30, 60, 90, and 120 trials) were entered as fixed effects, including their interaction. Participant ID was included as a random intercept to account for repeated observations within individuals and to model stable between-participant differences in baseline performance. To preserve participant anonymity, temporary participant identifiers (IDs) were assigned independently on each testing day. Consequently, participant identities could not be linked across testing sessions, and repeated measurements were modelled only within the same testing day. This approach allowed training-related effects to be estimated while accounting for baseline variability in perceptual judgments. Where appropriate, significant interactions were further explored using post hoc paired-sample t-tests with Holm–Bonferroni correction for multiple comparisons.

For the RDE model, arm length was additionally included as a covariate. Arm length was included because reachability estimates are inherently constrained by individual anthropometrics. Participants with longer arms can legitimately report larger reaching distances, independent of any task-related recalibration of perceived reachability. Controlling for arm length therefore reduces between-participant variance that reflects biomechanical capacity rather than perceptual change, and improves the interpretability and sensitivity of the RDE outcome in a single-group pre-post design.

Throughout the manuscript, the term *Trial Block* refers to a specific virtual tool-use training condition defined by the number of completed training trials (30, 60, 90, or 120 trials). The term *Session* refers to the assessment phase and distinguishes between the Pre-test and Post-test measurements.

### 3.1. Results

#### 3.1.1. Effects of tool-use training on perceived tactile distance depend on trial number.

We first tested whether participants showed systematic overestimation or underestimation of tactile distances at baseline. Errors were calculated as perceived minus actual distance, with positive values indicating overestimation. Baseline data are reported across the four stimulus distances (20, 40, 60, and 80 mm) and four blocks of tool-use training (30, 60, 90, and 120 trials) in Fig 4. Mean baseline errors ranged from 14.7 to 18.0 mm across blocks (SE = 1.47–1.66 mm), with individual responses spanning a wide range from −20.0 to 98.0 mm.

**Fig 4:**
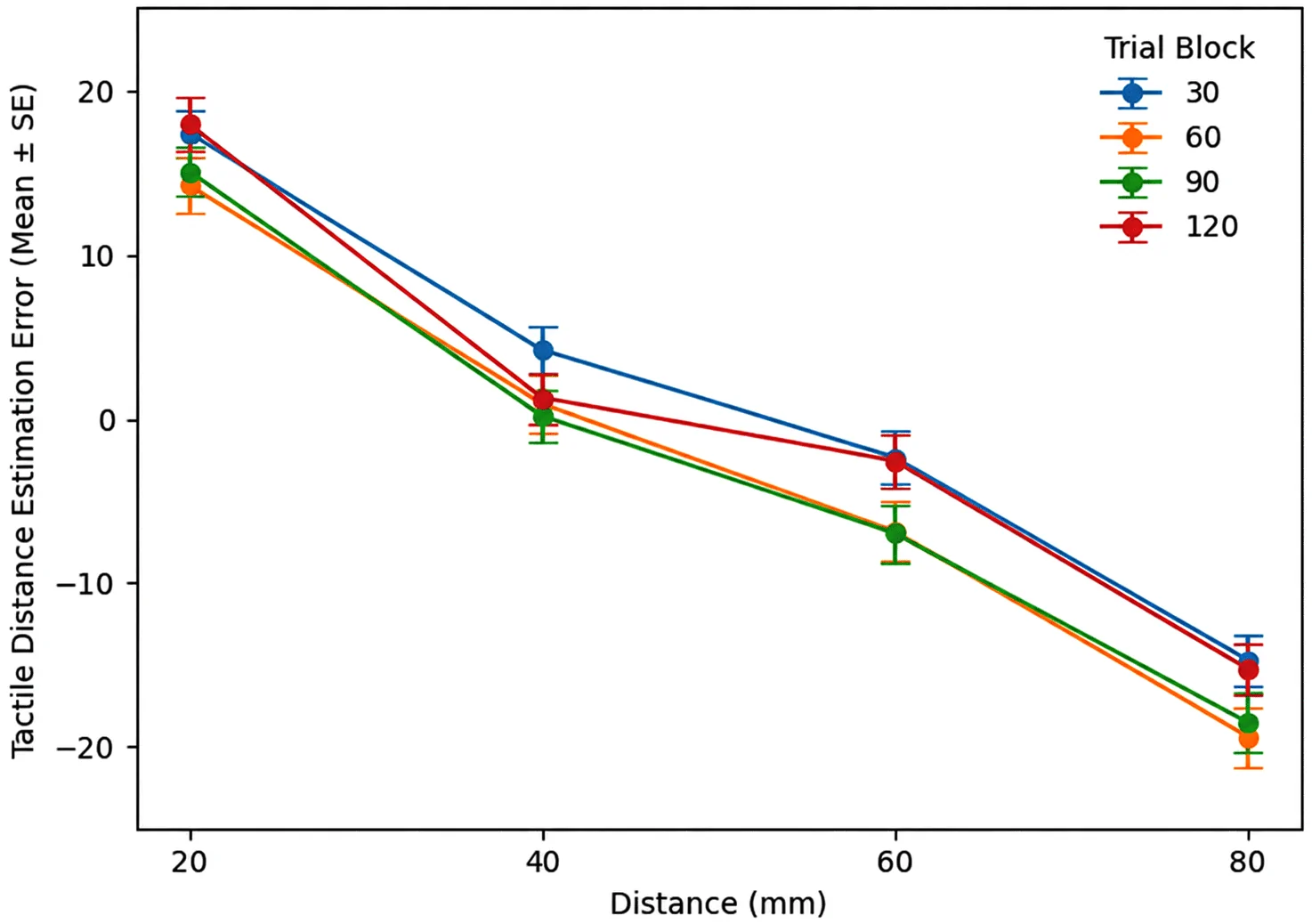
Baseline tactile distance estimation errors (TDJ) as a function of stimulus distance and experimental Trial Block. Lines represent mean tactile distance estimation error (± SE; N = 32) across participants for each stimulus distance (20, 40, 60, 80 mm) and Trial Block (30, 60, 90, 120 trials). Error bars denote the standard error of the mean. Negative values indicate underestimation of tactile distance.

Overall, at baseline the tactile distance estimation revealed a clear stimulus distance-dependent bias, characterised by strong overestimation at small distances, near-veridical perception at intermediate distances, and increasing underestimation as stimulus distance increased.

At 40 mm, mean baseline errors were close to veridical, with mean values ranging from 0.38 to 4.40 mm across blocks (SE = 1.52–1.72). At 60 mm, baseline performance shifted towards underestimation, with mean errors ranging from −2.29 to −7.28 mm (SE = 1.64–1.78). For the largest stimulus distance (80 mm), pronounced underestimation was observed. Mean errors ranged from −14.6 to −19.0 mm across trial blocks (SE = 1.58–1.89).

Baseline TDJ errors were analyzed using a linear mixed model with Stimulus Distance (20, 40, 60, 80 mm) and Trial Block (30, 60, 90, 120 trials) as factors, while controlling for Participant’s ID as random intercept. Baseline TDJ error differed across stimulus distances (F(3, 425) = 313.71, p < .001, ηp^2^ = .689) while the main effect of Trial Block was not significant (F(3, 425) = 1.80, p = .146, ηp^2^ = .013). Importantly, the Distance × Trial Block interaction was also not significant (F(9, 425) = 0.29, p = .977, ηp^2^ = .006). Thus, at baseline TDJ errors were dominated by stimulus distance and did not show evidence for a qualitatively different patterns across Trial Block.

To examine whether virtual tool-use training altered TDJ, estimation errors were analyzed as a function of Session (Pre-test vs. Post-test), Trial Block (30, 60, 90, and 120 trials), and Stimulus Distance (20, 40, 60, and 80 mm), while accounting for repeated observations within temporary participant IDs. Repeated measurements were modelled within each testing day, whereas comparisons between the four trial blocks should be interpreted cautiously because they cannot be treated as fully repeated measures across testing days.

The linear mixed-effects model revealed significant main effects of Session (F(1, 909) = 22.20, *p* < .001, ηp^2^ = .024), Trial Block (F(3, 909) = 5.16, *p* = .002, ηp^2^ = .017), and Stimulus Distance (F(3, 909) = 625.01, *p* < .001, ηp^2^ = .673).

Importantly, the Session × Trial Block interaction was significant (F(3, 909) = 4.47, *p* = .004, ηp^2^ = .015), suggesting that the magnitude of the pre- to post-test change differed across training blocks (Fig 5). Although statistically significant, the interaction was small in magnitude. Neither the Session × Distance interaction (F(3, 909) = 1.81, *p* = .144, ηp^2^ = .006), the Trial Block × Stimulus Distance interaction (F(9, 909) = 0.21, *p* = .993, ηp^2^ = .002), nor the three-way Session × Trial Block × Stimulus Distance interaction (F(9, 909) = 0.40, *p* = .933, ηp^2^ = .004), reached significance.

**Fig 5:**
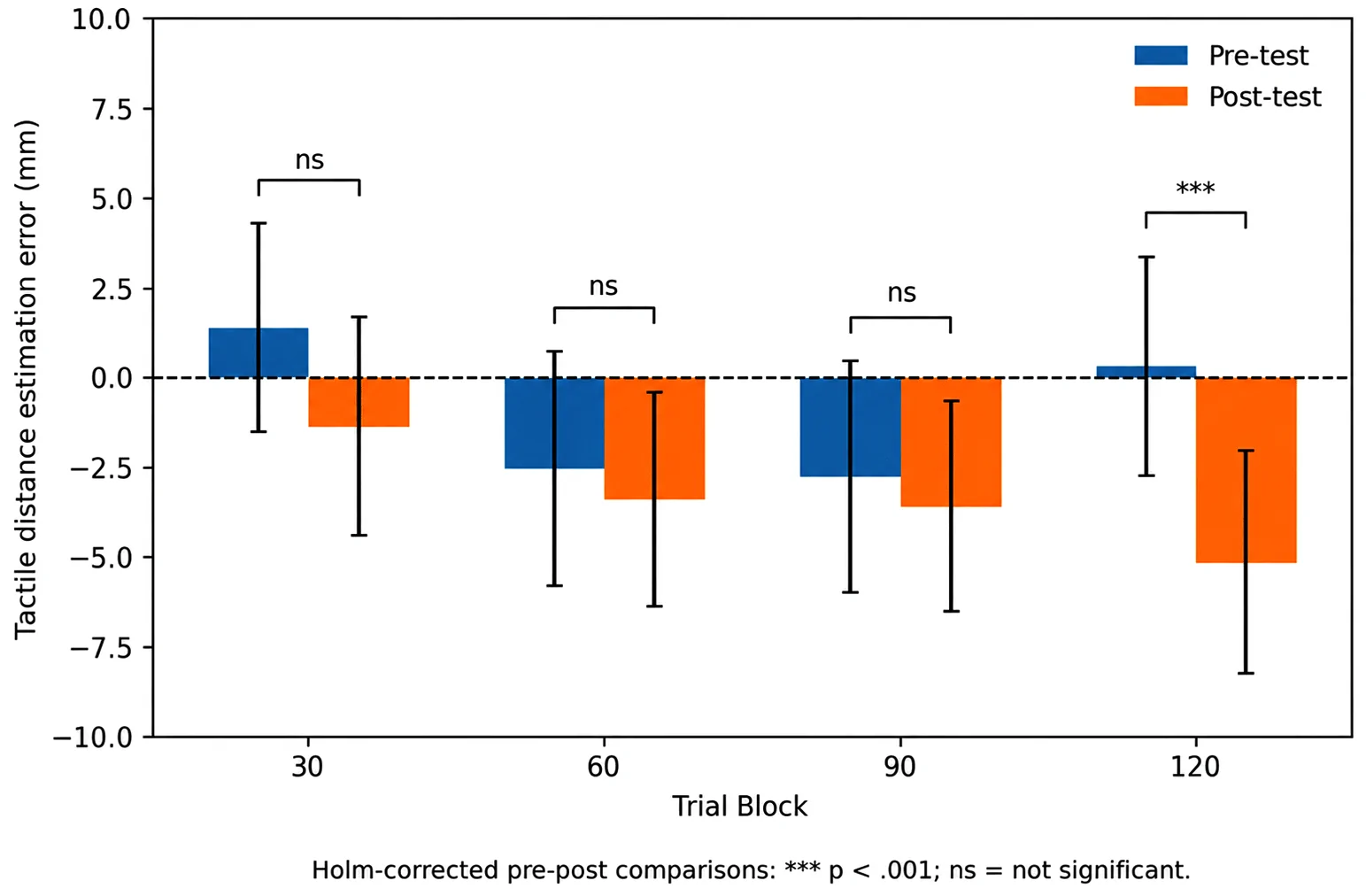
Tactile distance estimation (TDJ) error as a function of Session and Trial Block. Bars represent mean TDJ error (mm), collapsed across stimulus distances, for each Trial Block (30, 60, 90, 120 trials) separately for Pre-test and Post-test sessions. Error bars denote the standard error of the mean (SE; N = 32). Negative values indicate underestimation of tactile distance. Note: ***p < .001.

To identify which training blocks contributed to the significant Session × Trial Block interaction observed in the mixed-effects model, TDJ errors were averaged across stimulus distances for each participant ID and paired-samples *t*-tests comparing Pre-test and Post-test performance were conducted separately for each training block. Holm–Bonferroni correction was applied across the four comparisons. Following 30 trials, TDJ error decreased by 2.60 mm (95% CI [−5.53, 0.33]), although this reduction did not survive Holm correction (*t*(33) = −1.81, *p*Holm = .240, Cohen's *d* = 0.31). Following 60 trials, the mean change was −0.60 mm (95% CI [−3.94, 2.73]), which was not significant (*t*(26) = −0.37, *p*Holm = 1.000, Cohen's *d* = 0.07). Similarly, following 90 trials, the mean reduction was −0.84 mm (95% CI [−3.38, 1.69]), which was also not significant (*t*(30) = −0.68, *p*Holm = 1.000, Cohen's *d* = 0.12). In contrast, following 120 trials, TDJ error decreased by 5.66 mm (95% CI [−8.12, −3.20]), representing a large and statistically significant effect (*t*(31) = −4.69, *p*Holm < .001, Cohen's *d* = 0.83). Collectively, these participant-level comparisons indicate that the most robust evidence for tool-use–related recalibration of perceived forearm tactile distance emerged following 120 trials of virtual tool-use practice.

To determine whether the magnitude of changes in TDJ varied systematically across training blocks, participant-level change scores (Post-test − Pre-test) were calculated after averaging across stimulus distances and analysed with Trial Block as a fixed effect. This conservative analysis did not reveal a significant effect of Trial Block (F(3, 58) = 1.25, *p* = .301, ηp^2^ = .061).

Because participant identities could not be linked across testing days, this analysis effectively compared independent participant-block observations across the four training conditions rather than repeated measurements from the same individuals. Consequently, it had lower statistical power than a fully repeated-measures analysis and should be regarded as a sensitivity analysis rather than the primary test of training effects.

The psychophysical regression analysis revealed a significant reduction in intercept values following tool-use training, indicating a decrease in overall response bias (Pre-test: *M* = 25.40 mm; Post-test: *M* = 20.78 mm; Mean Difference = −4.88 mm, *t*(62) = −4.99, *p* < .001, Cohen's *d* = 0.63). Critically, perceptual gain also increased significantly from Pre-test to Post-test, with slope estimates increasing from 0.474 to 0.518 (Mean Difference = 0.047, *t*(62) = 2.86, *p* = .006, Cohen's *d* = 0.36). Thus, *t*he analysis directly compared the regression slopes between sessions and demonstrated that perceived forearm tactile distances tracked actual stimulus distances more closely following training. Together, these findings suggest that the observed reduction in TDJ error cannot be explained solely by a generic response bias. Rather, tool-use training modified the mapping between physical and perceived forearm tactile distance, providing evidence for a recalibration of forearm body representations. The simultaneous reduction in response bias and increase in perceptual gain further suggest that training influenced both the overall calibration and the fidelity of forearm tactile distance perception.

In summary, virtual tool-use training produced a significant reduction in TDJ error. Although the omnibus mixed-effects model indicated a modest Session × Trial Block interaction, participant-level analyses demonstrated that only the 120-trial condition yielded a robust pre- to post-training improvement after correction for multiple comparisons. These findings provide the strongest evidence that substantial changes in perceived forearm tactile distance emerged following extended virtual tool-use practice, whereas evidence for systematic improvements following shorter training durations remained limited.

#### 3.1.2. Changes in reaching distance estimation depend on trial number.

We then examined whether participants showed systematic over- or underestimation of reaching distance at baseline and whether RDE changed from pre- to post tool-use training as a function of Trial Block. Estimation Errors were calculated as perceived minus actual reaching distance, controlling for Arm Length, with negative values indicating underestimation.

At baseline participants showed a consistent tendency to underestimate reaching distance across Trial Block at baseline, with very comparable mean errors prior to each Trial Block. The RDE error prior to 30 tool-use trials had a mean of −30.4 cm (SE = 1.39), with individual values ranging from −46.5 to −15.3 cm. Prior to 60 tool-use trials, the error was −31.3 cm (SE = 1.20) and individual values ranged from −42.0 to −16.3 cm at Pre-test. Prior to 90 tool-use trials, the mean RDE error was −31.2 cm (SE = 1.29) and values ranged from −44.5 to −15.8 cm at Pre-test. Finally, prior to 120 tool-use trials, the mean RDE error was −30.4 cm (SE = 1.39) and individual values ranged from −47.2 to −14.0 cm. See Fig 6 for RDE at baseline.

**Fig 6:**
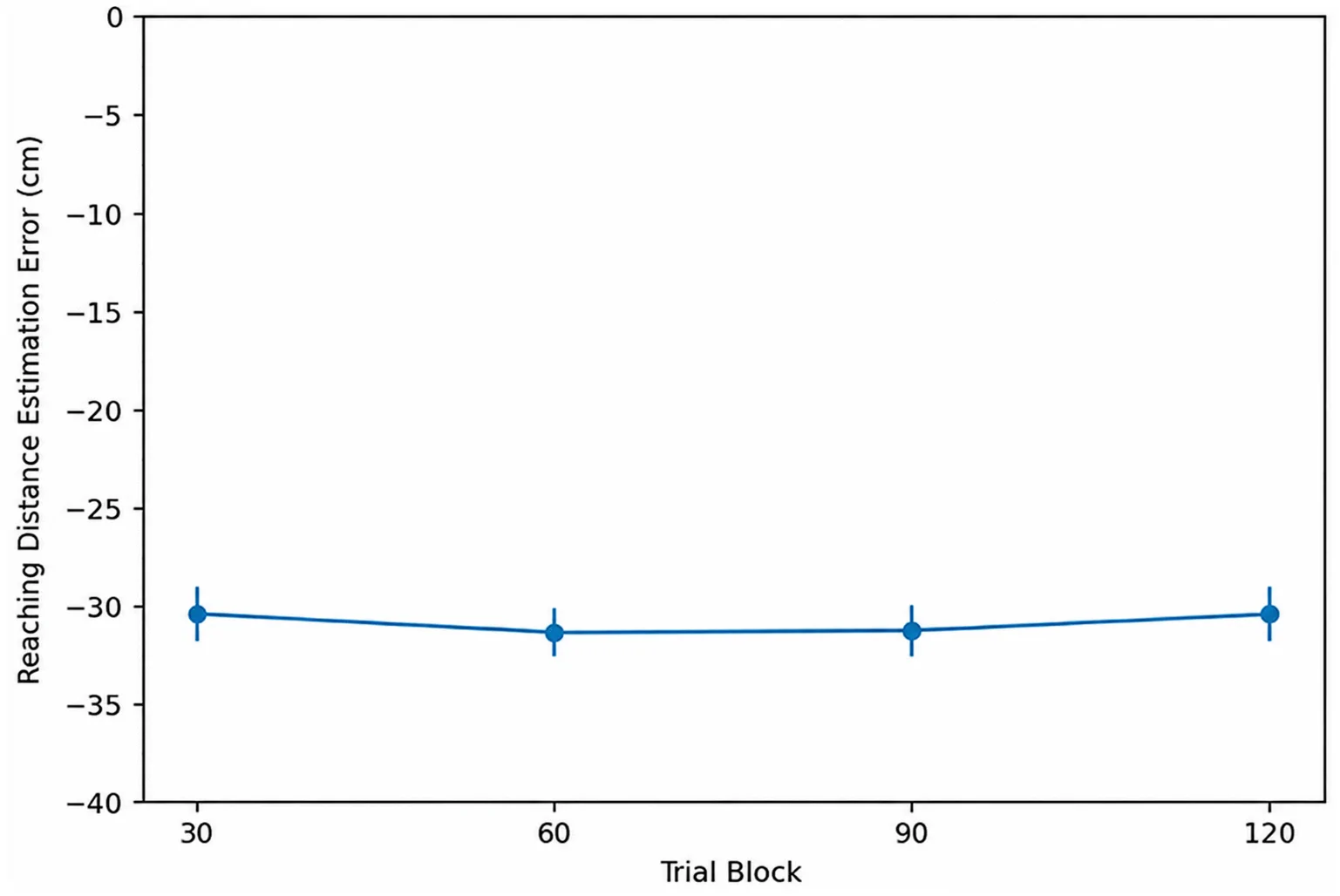
Baseline reaching distance estimation (RDE) error in cm as a function of Trial Block. Mean RDE error is shown for each Trial Block (30, 60, 90, and 120 trials) calculated relative to the participant’s arm length (mean ± SE, N = 32). Points indicate trial-specific means, and the connecting line is included to aid visualization across Trial Block.

To formally assess baseline stability in RDE, a general linear model was conducted on baseline (Pre-test) RDE errors with Trial Block (30, 60, 90, and 120 trials) as the factor. The analysis revealed no significant effect of Trial Block (F(3,128) = 0.15, p = .930, η^2^ = .004). Consistent with the visual impression from Fig 6, baseline RDE errors were highly similar across Trial Block (30 trials: M = −30.38 cm; 60 trials: M = −31.31 cm; 90 trials: M = −31.22 cm; 120 trials: M = −30.39 cm). These findings confirm a stable tendency to underestimate reaching distance at baseline, irrespective of the subsequent amount of tool-use training.

As for TDJ, we analyzed the RDE errors using a linear mixed model with Session (Post-test vs. Pre-test) and Trial Block (30, 60, 90, 120 trials) as fixed effects, including their interaction, and participant as a random intercept. The model explained a large proportion of variance overall (conditional R^2^ = 0.85), while fixed effects alone accounted for a small proportion of variance (marginal R^2^ = 0.31), indicating substantial inter-individual variability in RDE performance. This revealed a significant main effect of Session (*F*(1, 180) = 26.46, *p* < .001), with RDE errors becoming more negative from Pre-test to Post-test (*β* = −1.86, SE = 0.36, *p* < .001), indicating a modest increase in underestimation of reaching distance following tool-use training. In contrast, there was no main effect of Trial Block (*F*(3, 114) = 0.08, *p* = .97), and the Session × Trial Block interaction was not significant (*F*(3, 180) = 0.68, *p* = .56), suggesting that the magnitude of the training-related change in RDE error did not depend on the amount of practice received. Fig 7 illustrates RDE for Pre and Post-test based on each Trial Block.

**Fig 7:**
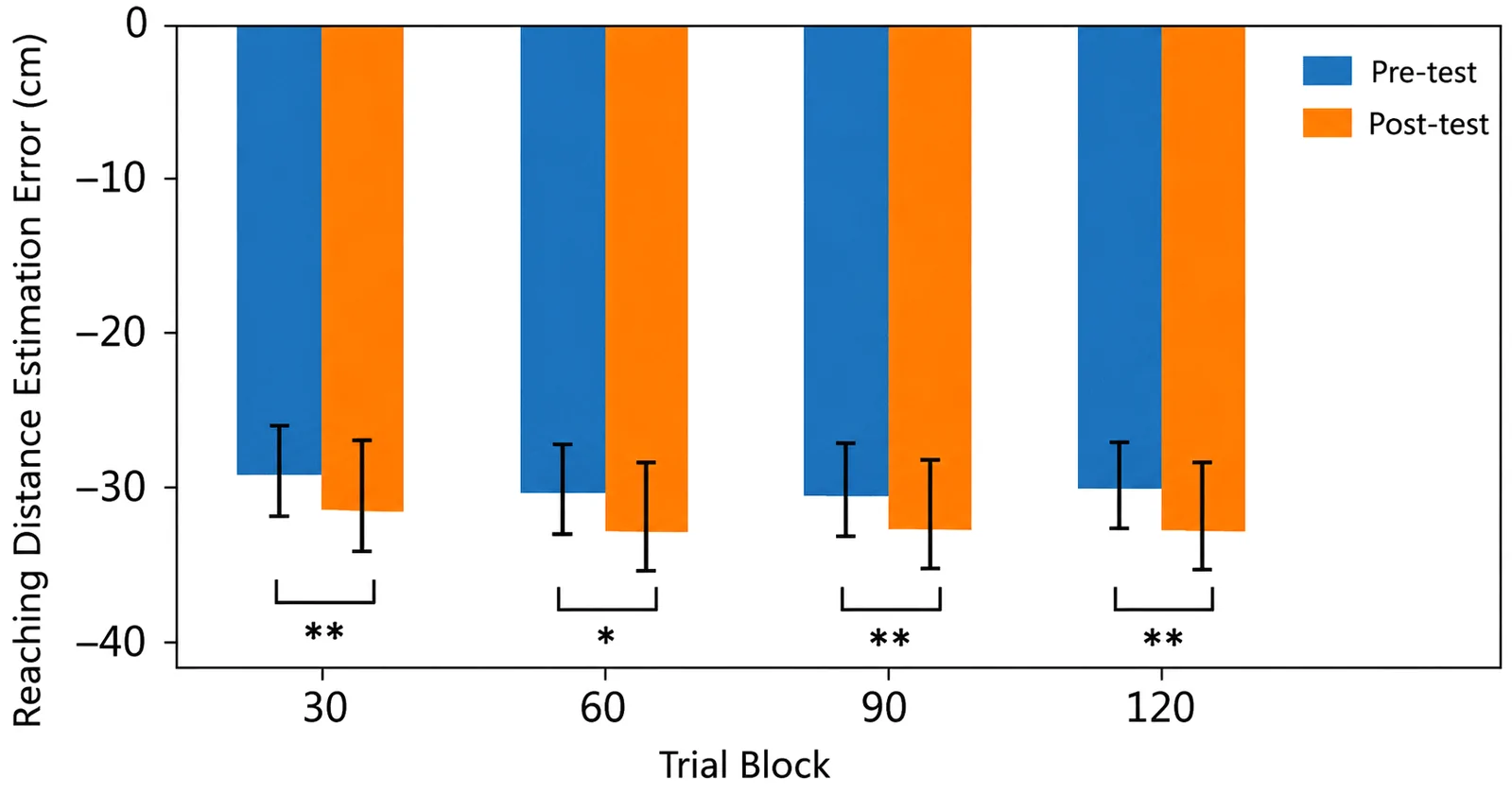
Reaching distance estimation (RDE) error in cm as a function of Session and Trial Block for N = 32. Bars show the mean RDE error (± SE), calculated relative to each participant’s arm length, for Pre-test and Post-test at each Trial Block (30, 60, 90, and 120 trials). Note: *p < .05, **p < .01.

Again, we directly compared the Pre- and Post-test errors for each Trial Block. Following tool-use training, underestimation increased slightly but reliably independently of the number of training trials. After 30 trials, RDE error was significantly larger at post-test (Mean Difference = −2.43 cm, *t*(32) = 2.95, *p* = .006, Cohen’s d = −0.51, 95% CI [−4.11, −0.76]), reflecting a shift toward greater underestimation. A similar pattern was observed after 60 trials, where post-test RDE error was slightly more negative than pre-test (Mean Difference = −1.34 cm, t(29) = 2.11, p = .044, Cohen’s d = −0.39, 95% CI [−2.63, −0.04]), and following 90 trials (Mean Difference = −1.77 cm), t(33) = 3.17, p = .003, Cohen’s d = −0.54, 95% CI [−2.91, −0.63]. After 120 trials, a reliable shift toward underestimation was observed was observed (Mean Difference = −2.03 cm), t(29) = 3.00, p = .006, Cohen’s d = −0.55, 95% CI [−3.42, −0.65].

Arm Length, entered to the model as a covariate, was a significant predictor of RDE error (F(1, 60) = 29.97, p < .001), with longer Arm Length associated with more negative error values (β = −0.69, SE = 0.13, t(60) = −5.47, p < .001), indicating that individual anthropometrics systematically shaped their RDE error values. Because RDE error was computed as estimated reachability minus actual Arm Length, more negative values reflect a larger underestimation of one’s reachable distance relative to the true Arm Length. Thus, participants with longer arms tended to underestimate their reach more strongly, suggesting that the perceptual “reach map” did not scale perfectly with body size. No interactions involving Arm Length were significant, including Trial Block × Arm Length (F(3, 111) = 0.61, p = 0.61), Session × Arm Length (F(1, 180) = 0.033, p = 0.85), and the three-way Trial Block × Session × Arm Length interaction (F(3, 180) = 0.29, p = 0.83). Finally, the intercept was significantly negative (β = −31.72, SE = 0.72, t(60) = −44.24, p < .001), indicating a robust baseline underestimation of reaching distance across participants. Importantly, the magnitude of this change was similar across Trial Block, suggesting that RDE was altered rapidly by tool-use and did not scale with additional practice. These results indicate that, unlike tactile distance estimation, reaching distance perception did not improve with extended training; instead, participants’ baseline underestimation of reachable space was maintained or slightly amplified following tool-use practice (cf., Fig 7). Across all experimental blocks, post-test means were consistently more negative than pre-test means, indicating that the increase in underestimation reflected a consistent shift across participants.

## 4. Discussion

In the present study, we examined whether different amounts of virtual tool-use training produce dissociable changes in perceived forearm tactile distance and perceived reaching distance. Previous work has shown that tool-use can alter body representation, but the role of training duration in shaping these effects has remained unclear. We therefore compared TDJ and RDE before and after virtual tool-use training across four Trial Block as practice doses (30, 60, 90, and 120 trials), and tested whether changes induced in a virtual training context transferred to subsequent physical assessment. The results revealed different temporal profiles for the two measures. TDJ showed an overall reduction in tactile distance estimation error following tool-use, with the most robust evidence for recalibration emerging after extended practice, here after 120 trials. In contrast, RDE changed from pre-test to post-test but did not vary with the number of tool-use trials. This pattern suggests that virtual tool-use can change forearm tactile distance, but that RDE is updated more rapidly and with less dependence on additional practice. Together, these findings support a dissociation between the two constructs, body representations and action-oriented estimates of reachability.

### 4.1. Practice-dependent recalibration of perceived forearm tactile distance

The TDJ findings indicate that virtual tool-use training can recalibrate the perceived tactile metric of the forearm (see, e.g., 6). At baseline, participants showed a distance-dependent pattern of tactile distortion: smaller stimulus distances were overestimated, intermediate distances were judged closer to veridically, and larger distances were underestimated. Such systematic distortions are consistent with the view that tactile distance perception depends not only on the physical distance between points on the skin, but also on an implicit representation of body form and body surface geometry [20,55]. Following tool-use, TDJ errors were reduced, consistent with previous evidence that tool-use can modify forearm tactile distance perception and body-related spatial representations [6,14,15,17–19].

The interpretation of reduced TDJ error requires some caution because the relation between perceived tactile distance and represented body size is theoretically debated. Miller and colleagues [20] distinguished between two possible mappings between tactile perception and internal body representation. According to an inverse model, expansion or lengthening of the represented limb could make two points on the skin correspond to a smaller relative fraction of the represented body surface, leading them to feel closer together. According to a proportional model, reduced perceived tactile distance would instead reflect a contraction or short-and-squat reshaping of the represented limb [55]. The present behavioural data cannot definitively distinguish between these alternatives. However, both accounts imply that systematic changes in TDJ reflect modifications in the mapping between physical stimulation and the internal representation of the limb, rather than a simple change in explicit response strategy.

This interpretation was strengthened by the complementary psychophysical regression analysis. Tool-use training reduced the intercept of the estimated-distance function, indicating a decrease in general response bias, but also increased the slope, indicating that perceived tactile distance tracked actual stimulus distance more closely after training. This pattern suggests that the TDJ changes cannot be explained solely by a nonspecific response shift. Instead, the data provide evidence for altered metric mapping between physical and perceived tactile distance, consistent with recalibration of forearm distance perception.

Importantly, the simultaneous reduction in response bias and increase in perceptual gain suggests that the observed changes in the forearm tactile distance perception measured by TDJ were not solely driven by a general shift in response tendencies. Rather, tool-use training altered how perceived tactile distance scaled with physical stimulus distance, providing additional evidence for plastic changes in forearm tactile representations.

The strongest and most reliable TDJ change emerged after extended practice. Although shorter training doses produced some numerical changes, the only significant evidence for recalibration was observed after 120 trials. This pattern suggests that updating body representation may require sustained exposure to the novel sensorimotor contingencies introduced by the virtual tool. Such gradual updating is consistent with the idea that perceived forearm tactile distance is constrained by the geometry and morphology of the biological body and may therefore require stronger or more consistent evidence before being recalibrated [20,28]. The use of virtual tool-use training is also theoretically informative. In the present paradigm, participants received systematic visual-motor feedback but no direct physical haptic feedback from the virtual tool. Nevertheless, changes in TDJ were observed during subsequent physical testing. This indicates that visual-motor contingencies alone can drive measurable changes in tactile body representation. At the same time, the need for extended practice before robust recalibration emerged suggests that the absence of direct tactile or haptic reinforcement may have slowed the updating process. This interpretation is consistent with accounts emphasizing the contribution of multisensory feedback to stable body representation plasticity [18,35,56], while also supporting the view that visual-motor information can be sufficient to induce plastic changes in body related perception [17,21,32,57].

A further implication concerns the transfer from virtual training to physical assessment. Because the tool-use experience occurred in a virtual environment, whereas TDJ was assessed physically, the observed effects cannot be reduced to a purely environment-specific response adaptation. Instead, the results suggest that virtual tool-use modified a representation that generalized beyond the immediate training context. This cross-context transfer supports the idea that virtual tool-use can engage sensorimotor mechanisms similar to those involved in real-world tool interactions [4,17,27,48]. More broadly, these findings indicate that virtual environments can provide a useful framework for isolating specific sensorimotor contingencies and testing their impact on body representation plasticity.

### 4.2. Practice-independent changes in reachable action space indexed by RDE

In contrast to the TDJ findings, changes in RDE were not practice-dependent. RDE errors became slightly more negative from pre-test to post-test, indicating a modest increase in underestimation of reachable distance following tool-use training. Crucially, this change was comparable across trial blocks and did not scale with the amount of practice. Thus, RDE appears to reflect a more rapid and generalized adjustment in perceived reachability rather than a gradual, dose-dependent recalibration.

This result should not be interpreted as evidence that RDE provides a direct measure of peripersonal space. Although peripersonal space and reachability space are related, they are not identical constructs. Successful interaction with an object requires estimating whether it can be reached directly by the body or through the use of a tool, making reachability judgments fundamentally concerned with perceived action capabilities. In contrast, peripersonal space is typically conceptualized as the multisensory space immediately surrounding the body and its effectors. Whereas peripersonal space is body-part centred and closely linked to multisensory integration, reachability space refers to the broader action-relevant region within which objects can be acted upon, depending on factors such as posture, limb length, tool-use possibilities, and current motor capacity [12,15,30]. Consequently, an object may be reachable without necessarily lying within the immediate multisensory space surrounding a specific body part. From this perspective, RDE is best understood as a behavioural index of perceived reachable action space rather than as a direct measure of peripersonal space boundaries.

This distinction helps interpret the practice-independent RDE pattern. Zanini and colleagues showed that peripersonal space and arm-reaching space can dissociate behaviourally and computationally [30]. While peripersonal space is closely tied to body-part-centred multisensory processing, reachability judgments can rely on a broader and more stable representation of action possibilities. The present RDE results are consistent with this architecture: once the tool successfully changes what the participant can do, perceived reachability may be updated rapidly, without requiring the same amount of repeated evidence as tactile body metrics.

Nevertheless, the RDE findings should be interpreted cautiously. Because the earliest post-training measurement occurred after 30 trials, the present design cannot determine whether reachability recalibration occurred immediately, after only a few successful actions, or gradually within the first 30 trials. Previous work has shown that tool-use can alter peripersonal and action-related spatial processing after relatively brief interaction periods [5,58]. Therefore, the absence of a dose-response relationship in the present data should not be taken to mean that practice plays no role in RDE changes. Rather, it suggests that reachable action space may adapt more rapidly than forearm tactile distance perception, reaching a functional asymptote within a short period of sensorimotor experience.

The dissociation between TDJ and RDE aligns with the idea that different representational systems impose different constraints on tool embodiment [20]. Forearm tactile distance perception is closely tied to the morphology and metric structure of the biological limb and therefore may require sustained sensorimotor evidence before being updated. In contrast, reachability judgments are action-oriented and may be more sensitive to immediate changes in functional capability. Tool-use therefore does not uniformly alter all spatial representations. Instead, it appears to affect forearm tactile distance perception according to their distinct computational roles and plastic limits.

### 4.3. Limitations and future directions

Several limitations should be acknowledged. First, the virtual tool-use paradigm relied primarily on visual-motor feedback and did not provide direct haptic resistance or tactile feedback. This design allowed us to isolate the contribution of visual-motor contingencies, but it also limits conclusions about how multisensory feedback would influence the speed and stability of recalibration. Future work should directly compare visual-only, haptic, and combined multisensory tool-use conditions to determine whether additional somatosensory feedback reduces the amount of practice required to modify tactile body representations.

Second, all post-training measurements were taken immediately after tool-use. Therefore, the present study cannot determine whether the observed changes reflect short-lived recalibration or longer-lasting representational plasticity. Longitudinal designs with delayed post-tests are needed to determine whether extended virtual tool-use produces stable changes that persist beyond the immediate post-training period.

Third, the study focused on healthy young adults, a single limb, and a specific virtual tool-use task. Generalization to other populations, tool geometries, effectors, or rehabilitation contexts should therefore be made cautiously. Future studies could examine whether similar practice-dependent and practice-independent patterns emerge in older adults, clinical groups, or tool-use tasks involving different levels of agency, embodiment, or functional relevance.

Fourth, combining these behavioural measures with neuroimaging, neurophysiology, or computational modelling would provide a stronger mechanistic account of the dissociation between TDJ and RDE. Such approaches could test whether forearm tactile distance recalibration and reachable action-space updating depend on different learning rates, different sensory weights, or partially distinct neural substrates.

## 5. Conclusion

The present study shows that virtual tool-use training can modify both forearm tactile distance perception and perceived RDE, but with different temporal profiles. TDJ provided evidence for practice-dependent recalibration of forearm tactile perception, with the strongest and most reliable effect emerging after extended practice. RDE, in contrast, changed from pre-test to post-test but did not depend on training dose, suggesting a rapid adjustment in perceived reachability. These findings support a distinction between perceived forearm tactile distance and estimates of reachable space. They further suggest that virtual tool-use can induce changes that transfer to physical assessment, providing a useful platform for studying body representation plasticity and tool embodiment.
