# Supplementary material for: Practice-dependent changes in body representation but not in reaching distance following virtual tool-use
Source: PLoS One. 2026 Oct 27;21(10):e0361297. doi: 10.1371/journal.pone.0361297 (PMC13712681; doi:10.1371/journal.pone.0361297)
Supplement: Supplementary file 1 [file pone.0361297.s002.docx]

**Response Letter**

2. Thank you for stating the following financial disclosure:

[The author(s) received no specific funding for this work.].

At this time, please address the following queries:

a) Please clarify the sources of funding (financial or material support) for your study. List the grants or organizations that supported your study, including funding received from your institution. Please also include the statement “There was no additional external funding received for this study.” in your updated Funding Statement.

Clarified.

b) State what role the funders took in the study. If the funders had no role in your study, please state: “The funders had no role in study design, data collection and analysis, decision to publish, or preparation of the manuscript.”

Clarified.

c) If any authors received a salary from any of your funders, please state which authors and which funders.

Not applicable.

d) If you did not receive any funding for this study, please state: “The authors received no specific funding for this work.”

Clarified.

Please include your amended statements within your cover letter; we will change the online submission form on your behalf.

**Funding Statement**

The authors gratefully acknowledge the financial support provided by the Faculty of Biology at Bielefeld University through the “Kleinforschung 2024'' grant awarded to Amir Jahanian Najafabadi, which enabled this research. The funders had no role in study design, data collection and analysis, decision to publish, or preparation of the manuscript. The authors received no specific funding for this work. There was no additional external funding received for this study.

3. Thank you for stating the following in your manuscript:

[The authors gratefully acknowledge the financial support provided by the Faculty of Biology at Bielefeld University through the ``Kleinforschung 2024'' grant awarded to Amir Jahanian Najafabadi, which enabled this research.]

We note that you have provided funding information that is not currently declared in your Funding Statement. However, funding information should not appear in the Acknowledgments section or other areas of your manuscript. We will only publish funding information present in the Funding Statement section of the online submission form.

Please remove any funding-related text from the manuscript and let us know how you would like to update your Funding Statement. Currently, your Funding Statement reads as follows:

[The author(s) received no specific funding for this work.]

Clarified.

Please include your amended statements within your cover letter; we will change the online submission form on your behalf.

**Funding Statement**

The authors gratefully acknowledge the financial support provided by the Faculty of Biology at Bielefeld University through the “Kleinforschung 2024'' grant awarded to Amir Jahanian Najafabadi, which enabled this research. The funders had no role in study design, data collection and analysis, decision to publish, or preparation of the manuscript. The authors received no specific funding for this work. There was no additional external funding received for this study.

4. When completing the data availability statement of the submission form, you indicated that you will make your data available on acceptance. We strongly recommend all authors decide on a data sharing plan before acceptance, as the process can be lengthy and hold up publication timelines. Please note that, though access restrictions are acceptable now, your entire data will need to be made freely accessible if your manuscript is accepted for publication. This policy applies to all data except where public deposition would breach compliance with the protocol approved by your research ethics board. If you are unable to adhere to our open data policy, please kindly revise your statement to explain your reasoning and we will seek the editor's input on an exemption. Please be assured that, once you have provided your new statement, the assessment of your exemption will not hold up the peer review process.

Thank you for your request and advises. We have included link to the datasets within the created OSF project. We will also upload all datasets led to these results as soon as the manuscript is accepted for publication with no reservation. The following statement has been added to the manuscript:

### **Data Availability Statement (To Be Made Available Upon Publication)**

The data supporting the conclusions of this article has been made available on OSF.org upon publication by the authors, without undue reservation. Datasets can be accessed via <https://osf.io/e4ux6/metadata/osf> (<https://doi.org/10.17605/OSF.IO/E4UX6>)

5. PLOS requires an ORCID iD for the corresponding author in Editorial Manager on papers submitted after December 6th, 2016. Please ensure that you have an ORCID iD and that it is validated in Editorial Manager. To do this, go to ‘Update my Information’ (in the upper left-hand corner of the main menu), and click on the Fetch/Validate link next to the ORCID field. This will take you to the ORCID site and allow you to create a new iD or authenticate a pre-existing ID in Editorial Manager.

**ORCID IDs:**

Amir Jahanian Najafabadi: <https://orcid.org/0000-0002-9246-5141>

Christoph Kayser: <https://orcid.org/0000-0001-7362-5704>

6. Your ethics statement should only appear in the Methods section of your manuscript. If your ethics statement is written in any section besides the Methods, please move it to the Methods section and delete it from any other section. Please ensure that your ethics statement is included in your manuscript, as the ethics statement entered into the online submission form will not be published alongside your manuscript.

Thank you. We moved the full statement into the method section.

7. We note that Figures 2 and 3 include images of participants in the study.

As per the PLOS ONE policy ([http://journals.plos.org/plosone/s/submission-guidelines#loc-human-subjects-research](http://track.editorialmanager.com/CL0/http:%2F%2Fjournals.plos.org%2Fplosone%2Fs%2Fsubmission-guidelines%23loc-human-subjects-research/1/010f019e8c8d828e-a07b565c-a50d-4c73-bee3-41dca280aadb-000000/iClw5A7VU1Q4GZKFuye9ghOOeRelZ6uc1YCOpYAl1Ns=258)) on papers that include identifying, or potentially identifying, information, the individual(s) or parent(s)/guardian(s) must be informed of the terms of the PLOS open-access (CC-BY) license and provide specific permission for publication of these details under the terms of this license. Please download the Consent Form for Publication in a PLOS Journal ([http://journals.plos.org/plosone/s/file?id=8ce6/plos-consent-form-english.pdf](http://track.editorialmanager.com/CL0/http:%2F%2Fjournals.plos.org%2Fplosone%2Fs%2Ffile%3Fid=8ce6%2Fplos-consent-form-english.pdf/1/010f019e8c8d828e-a07b565c-a50d-4c73-bee3-41dca280aadb-000000/tk63CfHBCsDzp4kX0XPGneiSy-eKQJ1yeDqu0R4escw=258)). The signed consent form should not be submitted with the manuscript, but should be securely filed in the individual's case notes. Please amend the methods section and ethics statement of the manuscript to explicitly state that the patient/participant has provided consent for publication: “The individual in this manuscript has given written informed consent (as outlined in PLOS consent form) to publish these case details”.

Please respond by return e-mail with an amended manuscript. We can upload this to your submission on your behalf.

If you are unable to obtain consent from the subject of the photograph, please either instruct us to remove the figure or supply a replacement figure by return e-mail for which you hold the relevant copyright permissions and subject consents. In some cases, you may need to specify in the text that the image used in the figure is not the original image used in the study, but a similar image used for illustrative purposes only. We can make any changes on your behalf.

The consent is attached for Fig 3. Fig to is the first author and is consent for publication.

This statement has also been added in each figure caption: “The individual in this manuscript has given written informed consent to publish these case details.”

8. Please ensure that you refer to Figure 6 in your text as, if accepted, production will need this reference to link the reader to the figure.

Thanks, added.

If the reviewer comments include a recommendation to cite specific previously published works, please review and evaluate these publications to determine whether they are relevant and should be cited. There is no requirement to cite these works unless the editor has indicated otherwise.

**Reviewers' comments:**

Reviewer's Responses to Questions

**Comments to the Author**

1. Is the manuscript technically sound, and do the data support the conclusions?

The manuscript must describe a technically sound piece of scientific research with data that supports the conclusions. Experiments must have been conducted rigorously, with appropriate controls, replication, and sample sizes. The conclusions must be drawn appropriately based on the data presented.

Reviewer #1: Partly

Reviewer #2: Partly

2. Has the statistical analysis been performed appropriately and rigorously?

Reviewer #1: No

Reviewer #2: Yes

3. Have the authors made all data underlying the findings in their manuscript fully available?

The [PLOS Data policy](http://track.editorialmanager.com/CL0/http:%2F%2Fwww.plosone.org%2Fstatic%2Fpolicies.action%23sharing/1/010f019e8c8d828e-a07b565c-a50d-4c73-bee3-41dca280aadb-000000/_AeDPfuKnocDid5R66ZKhHB7vXrjmnYHb3hhUBmcZc4=258) requires authors to make all data underlying the findings described in their manuscript fully available without restriction, with rare exception (please refer to the Data Availability Statement in the manuscript PDF file). The data should be provided as part of the manuscript or its supporting information, or deposited to a public repository. For example, in addition to summary statistics, the data points behind means, medians and variance measures should be available. If there are restrictions on publicly sharing data—e.g. participant privacy or use of data from a third party—those must be specified.

Reviewer #1: No

Reviewer #2: No

4. Is the manuscript presented in an intelligible fashion and written in standard English?

PLOS ONE does not copyedit accepted manuscripts, so the language in submitted articles must be clear, correct, and unambiguous. Any typographical or grammatical errors should be corrected at revision, so please note any specific errors here.

Reviewer #1: Yes

Reviewer #2: Yes

5. Review Comments to the Author

Please use the space provided to explain your answers to the questions above. You may also include additional comments for the author, including concerns about dual publication, research ethics, or publication ethics. (Please upload your review as an attachment if it exceeds 20,000 characters)

**Reviewer #1:**

The main goals of the present study were to 1) determine the amount of tool-use training necessary to induce plasticity in arm representation and peri-personal space, and 2) whether tool-use practice in a virtual environment transferred to real-world perception of one’s body and surrounding space. Across two days, the authors gathered pre- and post-training tactile distance judgments (TDJs) and reaching distance estimations (RDEs) following blocks of training with a virtual grabber that were either 30, 60, 90, or 120 trials in length. The authors found that exposure to 30, 90, and 120 trials with the virtual grabber led to more negative tactile distance estimation errors and post compared to pre-testing, with the difference being most pronounced after 120 trials. In contrast, RDE was dependent on arm length and not influenced by tool-use training. The study is interesting, well written, and has a clear objective. However, it is difficult to disentangle whether number of tool-use trials is truly driving the effect on tactile distance estimation errors, or whether testing day is confounding the results. Since day 1 induced reliable effects, but day 2 had mixed effects (significant differences after 90 but not 60 trials), one alternative explanation could be that initial tool-use recalibrates sensorimotor representations of the arm, but subsequent tool-use can render these representations resistant to change. Fixed intercepts for either testing day (1, 2) or trial block order (1,2,3,4) could be added to the linear mixed model to account for this variability. Please see detailed comments below.

We thank you for your constructive, insightful, and encouraging feedback on our manuscript. We have addressed every comment systematically by updating our Linear Mixed Models (LMMs), conducting formal baseline stability tests, clarifying our terminologies, and expanding our discussion to include the alternative explanations you raised. Below is our point-by-point response detailing the specific changes made to the manuscript.

**Introduction**
1. Did the authors have specific hypotheses about expected effects?

**Response:** Yes. We have explicitly stated our targeted hypotheses at the end of the Introduction section. Based on the functional demands of body metrics versus action boundaries, we predicted a gradual, practice-dependent scaling for the tactile body map (TDJ) and an immediate, trial-independent adaptation for reaching distance estimation (RDE). Please see lines 209-2015.

**Methods**
2. Lines 155-161: Did the authors conduct a power analysis to determine sample size? If not, why 34 participants? How many men and women were included in the study? In the results, the authors mention that the data is from “34 participants who completed the full experiment”. How many were excluded / did not complete the full experiment?

**Response:** We have updated the Methods section to include these missing details. The sample consisted of 34. 32 out of 34 recruited participants completed the full experimental blocks; while two participants were excluded for data analyses due to incomplete data. We did not collect demographic data such as age or gender to adhere local ethical guidelines at Bielefeld University.

With regard to the sample size, the following has been added to the manuscript line 220-226:

“Sample size was estimated based on the tool-use effect reported by Miller et al. (2014), particularly the arm-shaped-tool/arm condition, where tactile distance perception changed significantly following tool-use. Reconstructing the paired-samples effect size from the reported statistics yielded a conservative effect size of approximately Cohen’s d =0.86. A two-tailed paired-samples power analysis with α=.05 indicated that 13, 17, and 20 participants would be required to achieve 80%, 90%, and 95% power, respectively. To allow for exclusions and to provide stable estimates across training-dose conditions, we therefore aimed to recruit at least 30–35 participants.”

3. Lines 204-206: Were day 1 and day 2 consecutive? Was the delay between the two sessions the same for all participants?

**Response:** The testing days were non-consecutive and separated by a gap of minimum one week based on participants’ availability. We have updated the text to explicitly state this, which served as a reliable behavioral washout period between testing weeks.

**The following has been added to the manuscript (lines 249-269):**

**“**To isolate the effects of training duration and minimize carry-over between blocks, a 30-minute rest period was imposed between the two practice blocks on the same day, in line with recent tool-use research (Jahanian Najafabadi et al., 2023b, 2023a; Jahanian Najafabadi, Rastegari, Imani, et al., 2026; Jahanian Najafabadi, Rastegari, & Longo, 2026; Jahanian Najafabadi & Godde, 2025). This washout interval was selected based on previous evidence indicating that tool-use–induced modulations of somatosensory cortical responses can persist for approximately 10 minutes following training (Miller et al., 2019a). In addition, the order of the two tool-use training blocks (e.g. 30 and 120) within each testing day was counterbalanced across participants to control for potential order effects. On Day 1, the blocks consisted of 30 and 120 trials; on Day 2, of 60 and 90 training trials.

To further reduce carry-over effects between testing days, the two days were separated by at least one week. Previous research has shown that somatosensory perceptual changes induced by brief periods of motor learning can remain detectable for at least 24 hours (Ostry et al., 2010). Although the persistence of virtual tool-use effects was not directly examined in the present study, this evidence suggests that training-induced sensorimotor adaptations may extend beyond the immediate post-training interval. The one-week separation was therefore implemented to minimize residual effects of prior training and increase the independence of the two testing days. Taken together, the washout interval, within each testing day counterbalancing (between two-blocks), and multi-day separation were implemented to minimize contamination between training conditions and ensure that observed changes could be attributed primarily to the experimental manipulations rather than residual effects of previous tool-use experience.**”**

**Results**
4. Lines 327-345: Did the authors run a formal statistical test to confirm that baseline testing involved differences in distance estimations at short, medium, and long distances? The results reported are descriptive. From Figure 4, it looks like errors were higher for the 30 and 120 trial blocks compared to 60 and 90 trial blocks, particularly at the 60mm distance. This could be influencing why differences in the TDJ task between pre-and post-testing following 60 trials was not significant in the linear mixed model.

**Response:** We thank the reviewer for this observation. To formally evaluate baseline differences, we conducted an LMM on pre-test TDJ errors with Stimulus Distance (20, 40, 60, and 80 mm) and Trial Block (30, 60, 90, and 120 trials) as factors.

The analysis revealed a significant main effect of Stimulus Distance (F(3,2524) = 281.87, p < .001, ηp² = .251), confirming that participants systematically overestimated short tactile distances and underestimated longer tactile distances at baseline. A significant main effect of Trial Block was also observed (F(3,2524) = 5.95, p < .001, ηp² = .007), indicating modest differences in overall baseline error magnitude across blocks, although the effect size is very small and should be interpreted with cautious. Importantly, the Stimulus Distance × Trial Block interaction was not significant (F(9,2524) = 0.30, p = .976, ηp² = .001), demonstrating that the distance-dependent pattern of tactile distortion was highly similar across all trial blocks. To further address the possibility that baseline differences at the 60-mm stimulus distance influenced subsequent training effects, follow-up comparisons were conducted. Although baseline underestimation was numerically greater in the 60-trial (M = −6.87 mm) and 90-trial (M = −6.99 mm) blocks than in the 30-trial (M = −2.37 mm) and 120-trial (M = −2.60 mm) blocks, these differences were small in magnitude (all Cohen's d ≈ 0.20) and did not reach statistical significance (all ps > .05). Thus, while minor baseline differences were present, there was no evidence that the 60-trial condition exhibited a qualitatively distinct pattern of tactile perception that could account for the absence of a significant pre-post change in that condition.

5. Lines 352-358: The 30 and 120 trial blocks were always on day one and 60 and 90 trial blocks were always on day 2. So, the 30/120 meant that participants always had less experience with the tool compared to 60 /90. Did the authors consider adding a fixed effect for testing day (1 or 2) or training block order (1-4). This could be a good idea especially since the fixed effects only accounted for a small proportion of the variance in the model.

**Response:** Thank you for your comment. We agree that a fully crossed design, in which all trial blocks were distributed across both testing days, would provide the strongest control for potential day-related influences. In the present study, however, trial blocks were counterbalanced within testing days rather than across days. Importantly, several observations suggest that the reported practice-dependent effects cannot be readily explained by a day confound. First, baseline analyses revealed no Trial Block × Distance interaction, suggesting that the pattern of tactile distance distortion was not very different across blocks. Second, if testing day were the primary driver of the observed effects, similar outcomes would be expected for the two blocks administered on the same day. This was not observed. Specifically, the 60-trial condition did not show a significant pre-post change, whereas the 90-trial condition, which was administered within the same testing session, did show a significant change. Likewise, the strongest effect emerged in the 120-trial condition. These findings suggest that the observed differences cannot be attributed solely to day-related variability and are more consistent with a practice-dependent influence of tool-use training on tactile distance perception.

We nevertheless acknowledge that the partial confounding of trial block and testing day represents a limitation of the design and have added this point to the Discussion lines 751-754:

“Although testing sessions were separated by at least one week and block order was counterbalanced within each session, trial blocks were not fully crossed across testing days. Consequently, we cannot completely exclude the possibility that day-related variability contributed to some of the observed block differences.”

6. Line 373: “Reduction in estimation error” is a bit misleading. It sounds like participants became more accurate after tool-use training, when they were really underestimating differences. Please reword here and throughout the manuscript for clarity.

**Response:** We appreciate this correction. We have edited the manuscript and globally updated our terminology. Phrases such as "reduction in error" have been replaced with clear descriptors, such as a "shift toward greater tactile distance underestimation" or "post-test compression of perceived tactile distance," making it completely clear that the representation shifted rather than becoming objectively more accurate.

7. Lines 380-389: It seems the comparisons of TDJ estimation errors at post-test between different amounts of tool-use practice were evaluated with individual paired t-tests for each distance. Were corrections for number of t-tests performed applied? Why not opt for a more robust repeated measures ANOVA?

**Response:** We have clarified the text to reflect that our primary LMM structure operates as the modern, robust equivalent of a repeated-measures ANOVA, capable of handling repeated measures while accounting for individual random variations. The paired t-tests were strictly implemented as post-hoc decompositions following the significant omnibus interaction of the LMM. We have explicitly clarified that all post-hoc t-tests were controlled using a strict Holm-Bonferroni correction to prevent Type I error inflation.

8. Lines 386-389: The authors claim that the “findings suggest that tool-use induced changes in tactile distance perception scale with the amount of sensorimtotor experience, consistent with the gradual recalibration of body representation through extended tool-use”, but I’m not convinced this conclusion can be drawn definitively from the data. The recalibrations occurred at the shortest (30) and longest (120) trial blocks that happened to be on the same day, while the 60-trial block on day 2 did not lead to any pre- to post-testing differences. From this point of view, it seems that there could be an initial recalibration in body representation following novel tool-use that becomes resistant to change with further tool-use, which is why effects are not seen consistently on day 2 of testing. If the 60+90 trials and 30+120 trials had been counterbalanced so they were split across day 1 and day 2, I would find the authors claim more convincing. Including either day of testing (day 1, day 2) or trial block order (1,2,3,4) as fixed effects in the linear model could help strengthen the authors’ claim.

**Response:** We thank the reviewer for highlighting this critical alternative interpretation. It is completely true that because the 30/120 blocks and 60/90 blocks were grouped on separate days, testing day could act as a potential confound if the representation became "resistant to change" after the first session.

Please see our revised discussion section.

9. Figures 5 & 7: Indicate significant differences with asterisks.

**Response:** Done. Asterisks denoting the Holm-Bonferroni corrected significance values have been overlaid onto the bars in both Figure 5 and 7, and their definitions have been added to the figure legends.

10. Lines 402-409: I can see that the line in Fig 6. Is very flat so I believe that there was a “consistent tendency to underestimate reaching distance across all tool-use trial blocks at baseline”. It would be good to prove this statistically with an ANOVA for the sake of rigor.

**Response:** We thank the reviewer for this suggestion. To formally evaluate whether baseline reaching distance estimation differed across the four trial blocks, we conducted a general linear model on baseline (Pre-test) RDE errors. The analysis revealed no significant effect of Trial Number (F(3,128) = 0.15, p = .93, η² = .004). Baseline RDE errors were highly similar across all conditions (means ranging from −30.38 cm to −31.31 cm), confirming that participants exhibited a consistent tendency to underestimate reaching distance prior to tool-use training. We have added this analysis to the revised manuscript lines 561-567:

“To formally assess baseline stability in RDE, a general linear model was conducted on baseline (Pre-test) RDE errors with Trial Block (30, 60, 90, and 120 trials) as the factor. The analysis revealed no significant effect of Trial Block (F(3,128) = 0.15, p = .930, η² = .004). Consistent with the visual impression from Figure 6, baseline RDE errors were highly similar across Trial Block (30 trials: M = −30.38 cm; 60 trials: M = −31.31 cm; 90 trials: M = −31.22 cm; 120 trials: M = −30.39 cm). These findings confirm a stable tendency to underestimate reaching distance at baseline, irrespective of the subsequent amount of tool-use training.”

11. Lines 415-417: The methods section mentions that arm length was included as a covariate but is not reported in the model. Please clarify.

**Response:** We apologize for this oversight in the text. The continuous covariate of Arm Length was fully included in the LMM and was highly significant (F(1, 60) = 29.97, p < .001, β = -0.69, SE = 0.13, t(60) = -5.47, p < .001). We have explicitly written these complete model metrics into the revised Results text line 594 onwards.

12. Lines 428-436: Comment 7 above also applies to this analysis. Isn’t an ANOVA with follow-up t-tests is more appropriate? At the least, corrections for the number of t-tests performed should be applied.

**Response:** We thank the reviewer for this helpful suggestion. We agree that the training effects should first be evaluated using an omnibus analysis rather than through multiple uncorrected *t*-tests. In the revised manuscript, we therefore use a linear mixed-effects model as the primary omnibus analysis, with Session, Trial Block, Stimulus Distance, and their interactions entered as fixed effects and participant ID included as a random intercept. We chose a mixed-effects approach rather than a conventional repeated-measures ANOVA because it allows the repeated observations and participant-level variability to be modelled directly. Following the significant Session × Trial Block interaction, we conducted paired-samples *t*-tests comparing Pre-test and Post-test within each Trial Block only as follow-up analyses. To control the family-wise Type I error rate, these four comparisons were corrected using the Holm–Bonferroni procedure. Accordingly, the revised analysis addresses the reviewer’s concern by combining an omnibus test with multiplicity-corrected follow-up comparisons.

13. Lines 435-436: “After 120 trials, a decrease increase was observed”. Please fix.

**Response:** This typographical error has been corrected to read: "After 120 trials, a reliable shift toward underestimation was observed.

14. Line 437-438: Please state the statistical test performed at the beginning of this paragraph for clarity (e.g., We performed a 3-way mixed ANOVA ….). It’s confusing to include both the analysis of pre- and post-test RDE error across the tool-use trial lengths and this ANOVA. Please justify including both or just report the ANOVA since it seems like the consistent changes in RDE error from pre- to post-test across tool-use trials can be explained by differences in arm length, which is the main conclusion drawn from the results.

**Response:** We have added a clear opening statement to this section. We opted to use a Linear Mixed-Effects Model (which functions identically to a mixed ANCOVA) because it allows us to test the main effect of training session (Pre vs. Post) while simultaneously controlling for and reporting the continuous anatomical variance of Arm Length. This allows us to confirm that while an overall pre-to-post action-space shift occurred, the absolute scale of the error is inherently bound to individual physical arm metrics.

**Discussion**
15. The authors claim that the TDJ results indicate “gradual recalibration of perceived forearm tactile distance” based on the greatest change in errors from pre- to post-testing occurring at 120 trials is quite strong since the recalibrations only consistently happened on day 1 of testing. Once participants returned on day 2 after having had extended experience with the tool, the recalibration was less reliable. The authors provide a potential explanation based on representational competition (lines 493-496), but alternatively couldn’t day of testing be driving the effects (see comment 8)? If participants’ body representations become resilient to tool-use training, this is a key insight that needs to be followed up with dedicated studies. That the same configuration of trials occurred on each day across participants (30 + 120 trials always on day 1, and 60 + 90 trials always on day 2) should be mentioned in the limitations section.

**Response:** We have expanded our discussion to fully embrace this alternative viewpoint. In Section 4.1, we discuss how the initial tool exposure on Day 1 may have caused a strong representational calibration that made the body schema temporarily resilient or "resistant to change" on Day 2. Additionally, we have explicitly added this fixed multi-day trial distribution as a clear limitation in Section 4.3, noting that future research should fully cross block sequences across days to further isolate this phenomenon.

**Reviewer #2:**

In the present study, Najafabadi & Kayser investigate whether tool-induced changes in body representation are dependent upon the amount of exposure to the tool the user had. They used the TDJ and RDE to measure perception and action-oriented representations, respectively. These tasks were measured before and after performing a VR-based tool use task with four different lengths (30, 60, 90, and 120 trials). Tool use changed responses to the TDJ and RDE, consistent with plasticity in body representation. The TDJ effects were dependent upon how long the tool was used, whereas the RDE effects were not. This pattern of results is taken as evidence for practice-dependent effects in perceptual body representation but not action-oriented representation. The present study is interesting and addresses an often-ignored topic: How much tool use is needed to change body representation? The results would therefore be interesting to those in the field. However, there are several shortcomings with the study in its current form that prevent me from currently endorsing it for publication. I list these below.

**Major points**

1) Throughout the paper, a change in somatosensory perception (here, tactile distance) is taken as evidence for a shrinkage of the represented limb size. This interpretation is controversial and should be discussed as such. Whereas some have interpreted the change as a shrinkage (e.g., Miller et al., 2014), many interpret the change as an increase in represented size (e.g., Cardinali, Martel, Sposito, Garbarini, etc.). I do not personally think there is a definitive answer to this question, especially since it seems to depend on the paradigm being used (e.g, tactile localization, midpoint bisection, TDJ). I suggest the authors be a bit more cautious in their interpretation, especially when discussing prior research.

**Response:** Thank you for your comment. As suggested by reviewer, we extensively discussed this ongoing debate and integrated the shorter version of the following content into the discussion:

**“**In this research, the observed post-training reduction in TDJ overestimation is discussed as an implicit 'shrinkage' or contraction of the represented forearm length. However, this metric interpretation remains highly debated in the literature, where tool-use is frequently thought to induce a morphological *extension* or lengthening of the body schema to incorporate the tool’s physical dimensions (e.g., Cardinali et al., 2009a; Martel et al., 2019; Sposito et al., 2012). This apparent paradox can be resolved by applying the psychophysical frameworks outlined by Miller, Longo, and Saygin (2014), who distinguish between two competing models mapping tactile perception onto internal body maps.

According to the inverse model, an inverse relationship exists between representational size and perceived tactile distance: as a tool causes the underlying body representation to expand or lengthen, points of touch are remapped onto smaller relative anatomical fractions of that expanded representation, causing them to be perceived as closer together (i.e., a compression of tactile distance). Conversely, the proportional model (espoused by Longo & Haggard, 2011) dictates that tactile distance perception is strictly proportional to the internal map; thus, a reduction in perceived tactile distance directly reflects an actual metric 'shrinkage' or short-and-squat reshaping of the underlying representation.

While our behavioral data cannot definitively arbitrate between these inverse and proportional mechanisms, both frameworks demonstrate that a drop in TDJ overestimation indexes authentic representational plasticity rather than a generic response bias. Following Miller et al. (2014), we suggest that our results capture a highly localized, anisotropic shape modulation along the skin surface, which may serve as a functional adaptation to anchor the tool tip, rather than a definitive, global downsizing of the physical self-model. Given that these systematic distortions are observed in tactile tasks, these tool-induced modulations are best conceptualized as plastic updates to a primarily somatosensory, body-oriented tactile body model (Longo & Haggard, 2010), which operates independently of visual body images.**”**

2) It is not immediately clear to me how patterns of error relate to distortions of plasticity in spatial representation (e.g., arm length, pps). Indeed, I had to really think through what the direction of an error meant in terms of the numerical judgments. A more straightforward characterization of the behavior, in my opinion, is to use a regression approach to directly analyze the magnitude responses of each stimulus conditions (e.g., as Green 1982 did for the TDJ). Such an analysis would allow one to extract the bias in responses as well as the slope, which I would interpret as a gain that links perception (i.e., in tactile distance) with the underlying metric representation. As the current paper is now, a change in the error after tool use could be either due to a change in the spatial representation or a general change in response bias. I suggest that the authors also include this complementary approach, as it would provide a fuller picture of the data.

**Response:** We thank reviewer for this suggestion. We have conducted suggested analyses and added the following to the manuscript lines 373-386 and 527-539:

# **“Psychophysical Regression Analysis of TDJ**

To determine whether changes in TDJ performance reflected genuine modifications of tactile spatial representation rather than nonspecific response biases, a complementary psychophysical regression analysis was performed following Green (1982). For each participant and session, estimated tactile distance was regressed onto actual stimulus distance (20, 40, 60, and 80 mm) according to the following linear model:

Estimated Distance = *β0 + β1*(Actual Distance) + ε

where *β0* represents the intercept, reflecting a systematic response bias independent of stimulus distance, *β1* represents the slope, reflecting perceptual gain and the degree to which perceived distance scales with actual distance, and *ε* denotes residual error. In this framework, changes in the intercept indicate shifts in general response tendencies, whereas changes in the slope provide evidence for alterations in the underlying metric representation of tactile space. The analysis revealed a significant reduction in intercept values following tool-use training, indicating a decrease in overall response bias (Pre-test: *M* = 25.40 mm; Post-test: *M* = 20.78 mm; Mean Difference = −4.88 mm, *t*(62) = −4.99, *p* < .001, Cohen’s d = 0.63). Critically, perceptual gain also increased significantly from pre-test to post-test, with slope estimates increasing from 0.474 to 0.518 (Mean Difference = 0.047, *t*(62) = 2.86, *p* = .006, , Cohen’s d = 0.36). This increase in slope indicates that perceived tactile distances tracked actual stimulus distances more closely following training. Together, these findings suggest that the observed reduction in TDJ error cannot be explained solely by a generic response bias. Rather, tool-use training modified the mapping between physical and perceived tactile distance, providing evidence for a recalibration of body-centred spatial representations. The simultaneous reduction in response bias and increase in perceptual gain further suggest that training influenced both the overall calibration and the fidelity of tactile distance perception.**”**

**And the following has been integrated through the revised discussion:**

“This dissociation, whereby tactile body representations required cumulative practice to change whereas RDE adapted rapidly and independently of training duration, aligns closely with the representational constraints proposed by Miller et al. (2014). According to this framework, the necessary and sufficient conditions for tool embodiment differ across representational systems. Body-centred representations, such as the body schema and tactile body model, are tightly constrained by the geometry and morphology of the physical body. Consequently, updating these representations requires sufficient sensorimotor evidence to establish a reliable correspondence between the tool and the represented limb. This account is consistent with our TDJ findings, where substantial practice (120 trials) was required before robust and stable recalibration emerged corroborating research reported recently with a shorter virtual tool length (~ 30 cm) (Jahanian Najafabadi et al., 2023a).

In contrast, peripersonal space is not primarily concerned with representing the body itself, but rather with mapping the action-relevant space surrounding the body and its relationship to reachable objects (Brozzoli et al., 2013; Cardinali et al., 2009). Because peripersonal space is defined by action possibilities rather than strict morphological correspondence, its boundaries can be updated whenever a tool reliably alters the individual's functional capabilities. From this perspective, even brief successful interactions with a tool may be sufficient to modify perceived action boundaries. The practice-independent nature of the RDE findings supports this distinction. Whereas changes in tactile body representation appeared to require sustained sensorimotor experience, changes in perceived reachability emerged rapidly and remained relatively stable across training durations. Together, these findings suggest that body-centred and action-centred representations operate under different representational constraints, with the former exhibiting more conservative updating and the latter remaining highly responsive to changes in functional action capacity.”

3) I am not convinced that the current results, at least as presented, provide strong evidence for the presence of practice effects (in TDJ ) or their absence (in RDE). First, the authors assume that practice effects require a substantial number of trials (minimum 30) to accumulate towards a ceiling; hence their interpretation of the RDE task results. However, there is evidence that changes in PPS can happen almost immediately (see Holmes et al 2004). The authors cannot rule out practice effects in the RDE task that occur earlier than 30 trials. Second, from what I can tell, the different trial combinations (30+120, 60+90) were not counterbalanced across days. The baselines for the conditions on day 2 (60+90) look quite different than for day 1; can the authors rule out carry-over effects from the first day? Effects of motor learning can last several days, especially when the same task is involved. Third and relatedly, are the tool-use effects in the 60 and 90 trial conditions significantly different? Just eye-balling Figure 5 they look quite similar. Finally, are there carryover effects between the two conditions of the same day? There is EEG evidence that effects of tool use on somatosensory brain responses can last at least 30 minutes after tool use (e.g., Miller et al. 2019 JoCN). Any effect from one treatment condition may therefore carryover to the baseline of the other condition, making interpretation difficult.

**Response:** We thank reviewer for the thoughtful comment. We have revised our discussion accordingly. Please see the fully revised section.

4) The VR results of the current study have implications for the types of sensory or sensorimotor information that drives changes to body representation, which has been ongoing discussion in the literature. Whereas some have argued for an important role for visual feedback (e.g., visual illusions in Miller et al., 2017 and Guerraz et al., 2018) others have stressed the importance of somatosensory feedback (e.g., Martel et al., 2019; Miller et al., 2018, Bahmad et al., 2020). Readers would benefit from an in-depth discussion of this, as the current study has important implications for it.

**Response:** We thank the reviewer for this excellent recommendation. We agree that our virtual reality setup offers a unique, haptic-free environment that directly informs the ongoing debate regarding the sensory primitives (visual vs. somatosensory) driving body representation updates. We have expanded Section 4.1 of the Discussion to include a dedicated analysis of this topic. Specifically, we contrast accounts emphasizing visual feedback dominance (e.g., Miller et al., 2017; Guerraz et al., 2018) with those highlighting mandatory somatosensory loops (e.g., Miller et al., 2018; Martel et al., 2019; Bahmad et al., 2020). We position our study as an important bridge: it proves that visual and motor command signals alone are sufficient to cause cross-context transfer to physical representations, but suggests that the lack of haptic feedback may explain why a higher dosage (120 trials) is needed to achieve stable consolidation of the body schema.

Please see our revised discussion section.

**Minor points**

5) The way PPS is discussed feels a bit inconsistent to me. In section 1.1., the RDE is said to be a proxy for measuring PPS and also a global action-oriented representation of the body, which to me sounds a bit like body schema (and not PPS). Can the authors clarify what they mean by PPS and what RDE is thought to measure.

**Response:** We appreciate this important conceptual clarification. We agree that our initial phrasing conflated the boundary of peripersonal space with the internal mechanics of the body schema. We have revised Section 1.1 to explicitly disentangle these terms.

We now clearly state that the body schema is the plastic, somatosensory internal map of the body's physical dimensions, while peripersonal space represents the action-boundary map of the external space surrounding that limb. We clarify that the RDE task operationalizes the dynamic boundary of peripersonal space, but functionally relies on real-time inputs from both the body schema and body image (body metric representation) to calculate reachability. Therefore, RDE represents an action-oriented spatial judgment informed by body metrics, rather than a global structural body representation itself. We further expanded that section and discussed the differences across introduction section including lines 52-81.

6) I don’t follow the reasoning in the final sentence of the abstract, specifically this portion: “…the perceived metric of the limb itself appears to require cumulative sensorimotor evidence to overcome stable biological priors.” Significant effects were found at 30 trials. Though the magnitude of the results was cumulative, the term “overcome” suggests to me that there is a clear temporal point where the results go from no change to significant change. Please clarify this, in the abstract but also throughout the text where necessary.

**Response:** We agree with the reviewer’s observation and we revised the abstract.

6. PLOS authors have the option to publish the peer review history of their article ([what does this mean?](https://track.editorialmanager.com/CL0/https:%2F%2Fjournals.plos.org%2Fplosone%2Fs%2Feditorial-and-peer-review-process%23loc-peer-review-history/1/010f019e8c8d828e-a07b565c-a50d-4c73-bee3-41dca280aadb-000000/Vvv1e-tRD5mcDTrGh0-f2DUzMg6-z2x01OM1tolIjsE=258)). If published, this will include your full peer review and any attached files.

If you choose “no”, your identity will remain anonymous but your review may still be made public.

**Do you want your identity to be public for this peer review?** For information about this choice, including consent withdrawal, please see our [Privacy Policy](https://track.editorialmanager.com/CL0/https:%2F%2Fwww.plos.org%2Fprivacy-policy/1/010f019e8c8d828e-a07b565c-a50d-4c73-bee3-41dca280aadb-000000/fep3LWlpvfKCA4DuDAlNmQB6hF-r2T0UV9EN8zy2984=258).

Reviewer #1: No

Reviewer #2: No
