# Supplementary material for: Practice-dependent changes in body representation but not in reaching distance following virtual tool-use
Source: PLoS One. 2026 Oct 27;21(10):e0361297. doi: 10.1371/journal.pone.0361297 (PMC13712681; doi:10.1371/journal.pone.0361297)
Supplement: Supplementary file 2 [file pone.0361297.s003.docx]

**Response to Reviewers**

**Review Comments to the Author**

Please use the space provided to explain your answers to the questions above. You may also include additional comments for the author, including concerns about dual publication, research ethics, or publication ethics. (Please upload your review as an attachment if it exceeds 20,000 characters)

Reviewer #1: A wholehearted thank you to the authors for carefully considering each of my comments. I know it's painstaking work and I appreciate your open-mindedness and rigor. I have two outstanding points to address before I fully endorse the manuscript for publication.

**Response:** We highly appreciated your consideration and feedback improving our work.

First, PLOS One's policy is that the data be made fully available without restriction before publication, but the data availability statement reads that the data will be available on OSF.org only upon publication. Could the authors please share their data ahead of publication to conform to the journal's open science policies? See below for my remaining 2 concerns:

**Response:** Thank you for your comment. We have now uploaded datasets through OSF and revised the text accordingly.

3. Lines 204-206: Were day 1 and day 2 consecutive? Was the delay between the two sessions the same for all participants?

**Response:** The testing days were non-consecutive and separated by a gap of minimum one week based on participants’ availability. We have updated the text to explicitly state this, which served as a reliable behavioral washout period between testing weeks.

The following has been added to the manuscript (lines 249-269):
“To isolate the effects of training duration and minimize carry-over between blocks, a 30minute rest period was imposed between the two practice blocks on the same day, in line with recent tool-use research (Jahanian Najafabadi et al., 2023b, 2023a; Jahanian Najafabadi, Rastegari, Imani, et al., 2026; Jahanian Najafabadi, Rastegari, & Longo, 2026; Jahanian Najafabadi & Godde, 2025). This washout interval was selected based on previous evidence indicating that tool-use–induced modulations of somatosensory cortical responses can persist for approximately 10 minutes following training (Miller et al., 2019a). In addition, the order of the two tool-use training blocks (e.g. 30 and 120) within each testing day was counterbalanced across participants to control for potential order effects. On Day 1, the blocks consisted of 30 and 120 trials; on Day 2, of 60 and 90 training trials.
To further reduce carry-over effects between testing days, the two days were separated by at least one week. Previous research has shown that somatosensory perceptual changes induced by brief periods of motor learning can remain detectable for at least 24 hours (Ostry et al., 2010). Although the persistence of virtual tool-use effects was not directly examined in the present study, this evidence suggests that training-induced sensorimotor adaptations may extend beyond the immediate post-training interval. The one-week separation was therefore implemented to minimize residual effects of prior training and increase the independence of the two testing days. Taken together, the washout interval, within each testing day counterbalancing (between two-blocks), and multi-day separation were implemented to minimize contamination between training conditions and ensure that observed changes could be attributed primarily to the experimental manipulations rather than residual effects of previous tool-use experience.”

Reviewer Response: Thank you for adding the above details to the methods section. Can the authors be more specific than “at least one week”? What was the maximum number of days between sessions? What was the mean and standard deviation? If there is a lot of variation between participants, couldn’t this have impacted the results? Longer washout, less carry over effects?

**Response:** Thank you for your comment. As requested, we clarified and better phrased the exact detail in the respected section. The one-week period was ±1 day to make sure participants find a time to participate considering their student classes and their assignments.

**5. Lines 352-358:** The 30 and 120 trial blocks were always on day one and 60 and 90 trial blocks were always on day 2. So, the 30/120 meant that participants always had less experience with the tool compared to 60 /90. Did the authors consider adding a fixed effect for testing day (1 or 2) or training block order (1-4). This could be a good idea especially since the fixed effects only accounted for a small proportion of the variance in the model.

**Response:** Thank you for your comment. We agree that a fully crossed design, in which all trial blocks were distributed across both testing days, would provide the strongest control for potential day-related influences. In the present study, however, trial blocks were counterbalanced within testing days rather than across days. Importantly, several observations suggest that the reported practice-dependent effects cannot be readily explained by a day confound. First, baseline analyses revealed no Trial Block × Distance interaction, suggesting that the pattern of tactile distance distortion was not very different across blocks. Second, if testing day were the primary driver of the observed effects, similar outcomes would be expected for the two blocks administered on the same day. This was not observed. Specifically, the 60trial condition did not show a significant pre-post change, whereas the 90-trial condition, which was administered within the same testing session, did show a significant change. Likewise, the strongest effect emerged in the 120-trial condition. These findings suggest that the observed differences cannot be attributed solely to day-related variability and are more consistent with a practice-dependent influence of tool-use training on tactile distance perception.
We nevertheless acknowledge that the partial confounding of trial block and testing day represents a limitation of the design and have added this point to the Discussion lines 751-754:
“Although testing sessions were separated by at least one week and block order was counterbalanced within each session, trial blocks were not fully crossed across testing days. Consequently, we cannot completely exclude the possibility that day-related variability contributed to some of the observed block differences.”

**Reviewer Response:** Thank you for explaining your logic and including my comment in the discussion. I find the reasoning persuasive but would find the results of linear mixed model with either a fixed effect for testing day (1 or 2) or training block order (1-4) even more convincing. If the results are unchanged, this point wouldn’t even need to be mentioned as a limitation in the discussion. It would strengthen the results.

**Response:** Thank you for your comment. You are correct, we have now removed the related statement from limitation section.

**Minor comment:** p. 19, line 594: “entered to the model as covariate” ◊ “entered into the model as a covariate”

„a“ has been added.
