# Supplementary material for: Practice-dependent changes in body representation but not in reaching distance following virtual tool-use
Source: PLoS One. 2026 Oct 27;21(10):e0361297. doi: 10.1371/journal.pone.0361297 (PMC13712681; doi:10.1371/journal.pone.0361297)
Supplement: Peer review history

# Peer review: Decision Letter 0

**DOI:** 10.1371/journal.pone.0361297.r001

**Authors:** Patrick Bruns

3 Jun 2026

Dear Dr. Jahanian Najafabadi,

Thank you for submitting your manuscript to PLOS ONE. After careful consideration, we feel that it has merit but does not fully meet PLOS ONE’s publication criteria as it currently stands. Therefore, we invite you to submit a revised version of the manuscript that addresses the points raised during the review process.

Your manuscript has been reviewed by two experts in the topic. As you will see below, both reviewers had concerns about the interpretability of the findings due to potential carry-over effects from day 1 given that trial combinations (30+120, 60+90) were not counterbalanced across days. These concerns would need to be addressed in a revision, along with the additional comments from the reviews.

Please submit your revised manuscript by Jul 18 2026 11:59PM. If you will need more time than this to complete your revisions, please reply to this message or contact the journal office at plosone@plos.org. When you're ready to submit your revision, log on to https://www.editorialmanager.com/pone/ and select the 'Submissions Needing Revision' folder to locate your manuscript file.

- A letter that responds to each point raised by the academic editor and reviewer(s). You should upload this letter as a separate file labeled 'Response to Reviewers'.
- A marked-up copy of your manuscript that highlights changes made to the original version. You should upload this as a separate file labeled 'Revised Manuscript with Track Changes'.
- An unmarked version of your revised paper without tracked changes. You should upload this as a separate file labeled 'Manuscript'.

If you would like to make changes to your financial disclosure, please include your updated statement in your cover letter. Guidelines for resubmitting your figure files are available below the reviewer comments at the end of this letter.

If applicable, we recommend that you deposit your laboratory protocols in protocols.io to enhance the reproducibility of your results. Protocols.io assigns your protocol its own identifier (DOI) so that it can be cited independently in the future. For instructions see: https://journals.plos.org/plosone/s/submission-guidelines#loc-laboratory-protocols. Additionally, PLOS ONE offers an option for publishing peer-reviewed Lab Protocol articles, which describe protocols hosted on protocols.io. Read more information on sharing protocols at https://plos.org/protocols?utm_medium=editorial-email&utm_source=authorletters&utm_campaign=protocols.

As the corresponding author, your ORCID iD is verified in the submission system and will appear in the published article. PLOS supports the use of ORCID, and we encourage all coauthors to register for an ORCID iD and use it as well. Please encourage your coauthors to verify their ORCID iD within the submission system before final acceptance, as unverified ORCID iDs will not appear in the published article. *Only*  the individual author can complete the verification step; PLOS staff *cannot*  verify ORCID iDs on behalf of authors.

We look forward to receiving your revised manuscript.

Kind regards,

Patrick Bruns

Academic Editor

PLOS One

Journal Requirements:

When submitting your revision, we need you to address these additional requirements.

1. Please ensure that your manuscript meets PLOS ONE's style requirements, including those for file naming. The PLOS ONE style templates can be found at https://journals.plos.org/plosone/s/file?id=wjVg/PLOSOne_formatting_sample_main_body.pdf and https://journals.plos.org/plosone/s/file?id=ba62/PLOSOne_formatting_sample_title_authors_affiliations.pdf

2. Thank you for stating the following financial disclosure:

[The author(s) received no specific funding for this work.].

At this time, please address the following queries:

a) Please clarify the sources of funding (financial or material support) for your study. List the grants or organizations that supported your study, including funding received from your institution. Please also include the statement “There was no additional external funding received for this study.” in your updated Funding Statement.

b) State what role the funders took in the study. If the funders had no role in your study, please state: “The funders had no role in study design, data collection and analysis, decision to publish, or preparation of the manuscript.”

c) If any authors received a salary from any of your funders, please state which authors and which funders.

d) If you did not receive any funding for this study, please state: “The authors received no specific funding for this work.”

Please include your amended statements within your cover letter; we will change the online submission form on your behalf.

3. Thank you for stating the following in your manuscript:

[The authors gratefully acknowledge the financial support provided by the Faculty of Biology at Bielefeld University through the ``Kleinforschung 2024'' grant awarded to Amir Jahanian Najafabadi, which enabled this research.]

We note that you have provided funding information that is not currently declared in your Funding Statement. However, funding information should not appear in the Acknowledgments section or other areas of your manuscript. We will only publish funding information present in the Funding Statement section of the online submission form.

Please remove any funding-related text from the manuscript and let us know how you would like to update your Funding Statement. Currently, your Funding Statement reads as follows:

[The author(s) received no specific funding for this work.]

Please include your amended statements within your cover letter; we will change the online submission form on your behalf.

4. When completing the data availability statement of the submission form, you indicated that you will make your data available on acceptance. We strongly recommend all authors decide on a data sharing plan before acceptance, as the process can be lengthy and hold up publication timelines. Please note that, though access restrictions are acceptable now, your entire data will need to be made freely accessible if your manuscript is accepted for publication. This policy applies to all data except where public deposition would breach compliance with the protocol approved by your research ethics board. If you are unable to adhere to our open data policy, please kindly revise your statement to explain your reasoning and we will seek the editor's input on an exemption. Please be assured that, once you have provided your new statement, the assessment of your exemption will not hold up the peer review process.

5. PLOS requires an ORCID iD for the corresponding author in Editorial Manager on papers submitted after December 6th, 2016. Please ensure that you have an ORCID iD and that it is validated in Editorial Manager. To do this, go to ‘Update my Information’ (in the upper left-hand corner of the main menu), and click on the Fetch/Validate link next to the ORCID field. This will take you to the ORCID site and allow you to create a new iD or authenticate a pre-existing iD in Editorial Manager.

6. Your ethics statement should only appear in the Methods section of your manuscript. If your ethics statement is written in any section besides the Methods, please move it to the Methods section and delete it from any other section. Please ensure that your ethics statement is included in your manuscript, as the ethics statement entered into the online submission form will not be published alongside your manuscript.

7. We note that Figures 2 and 3 include images of participants in the study.

As per the PLOS ONE policy (http://journals.plos.org/plosone/s/submission-guidelines#loc-human-subjects-research) on papers that include identifying, or potentially identifying, information, the individual(s) or parent(s)/guardian(s) must be informed of the terms of the PLOS open-access (CC-BY) license and provide specific permission for publication of these details under the terms of this license. Please download the Consent Form for Publication in a PLOS Journal (http://journals.plos.org/plosone/s/file?id=8ce6/plos-consent-form-english.pdf). The signed consent form should not be submitted with the manuscript, but should be securely filed in the individual's case notes. Please amend the methods section and ethics statement of the manuscript to explicitly state that the patient/participant has provided consent for publication: “The individual in this manuscript has given written informed consent (as outlined in PLOS consent form) to publish these case details”.

Please respond by return e-mail with an amended manuscript. We can upload this to your submission on your behalf.

If you are unable to obtain consent from the subject of the photograph, please either instruct us to remove the figure or supply a replacement figure by return e-mail for which you hold the relevant copyright permissions and subject consents. In some cases, you may need to specify in the text that the image used in the figure is not the original image used in the study, but a similar image used for illustrative purposes only. We can make any changes on your behalf.

8. Please ensure that you refer to Figure 6 in your text as, if accepted, production will need this reference to link the reader to the figure.

If the reviewer comments include a recommendation to cite specific previously published works, please review and evaluate these publications to determine whether they are relevant and should be cited. There is no requirement to cite these works unless the editor has indicated otherwise.

[Note: HTML markup is below. Please do not edit.]

Reviewers' comments:

Reviewer's Responses to Questions

**Comments to the Author**

1. Is the manuscript technically sound, and do the data support the conclusions?

Reviewer #1: Partly

Reviewer #2: Partly

**********

2. Has the statistical analysis been performed appropriately and rigorously?

Reviewer #1: No

Reviewer #2: Yes

**********

3. Have the authors made all data underlying the findings in their manuscript fully available?

The PLOS Data policy

Reviewer #1: No

Reviewer #2: No

**********

4. Is the manuscript presented in an intelligible fashion and written in standard English?

Reviewer #1: Yes

Reviewer #2: Yes

**********

Reviewer #1: The main goals of the present study were to 1) determine the amount of tool-use training necessary to induce plasticity in arm representation and peri-personal space, and 2) whether tool-use practice in a virtual environment transferred to real-world perception of one’s body and surrounding space. Across two days, the authors gathered pre- and post-training tactile distance judgments (TDJs) and reaching distance estimations (RDEs) following blocks of training with a virtual grabber that were either 30, 60, 90, or 120 trials in length. The authors found that exposure to 30, 90, and 120 trials with the virtual grabber led to more negative tactile distance estimation errors and post compared to pre-testing, with the difference being most pronounced after 120 trials. In contrast, RDE was dependent on arm length and not influenced by tool-use training. The study is interesting, well written, and has a clear objective. However, it is difficult to disentangle whether number of tool-use trials is truly driving the effect on tactile distance estimation errors, or whether testing day is confounding the results. Since day 1 induced reliable effects, but day 2 had mixed effects (significant differences after 90 but not 60 trials), one alternative explanation could be that initial tool-use recalibrates sensorimotor representations of the arm, but subsequent tool-use can render these representations resistant to change. Fixed intercepts for either testing day (1, 2) or trial block order (1,2,3,4) could be added to the linear mixed model to account for this variability. Please see detailed comments below.

Introduction

1. Did the authors have specific hypotheses about expected effects?

Methods

2. Lines 155-161: Did the authors conduct a power analysis to determine sample size? If not, why 34 participants? How many men and women were included in the study? In the results, the authors mention that the data is from “34 participants who completed the full experiment”. How many were excluded / did not complete the full experiment?

3. Lines 204-206: Were day 1 and day 2 consecutive? Was the delay between the two sessions the same for all participants?

Results

4. Lines 327-345: Did the authors run a formal statistical test to confirm that baseline testing involved differences in distance estimations at short, medium, and long distances? The results reported are descriptive. From Figure 4, it looks like errors were higher for the 30 and 120 trial blocks compared to 60 and 90 trial blocks, particularly at the 60mm distance. This could be influencing why differences in the TDJ task between pre-and post-testing following 60 trials was not significant in the linear mixed model.

5. Lines 352-358: The 30 and 120 trial blocks were always on day one and 60 and 90 trial blocks were always on day 2. So, the 30/120 meant that participants always had less experience with the tool compared to 60 /90. Did the authors consider adding a fixed effect for testing day (1 or 2) or training block order (1-4). This could be a good idea especially since the fixed effects only accounted for a small proportion of the variance in the model.

6. Line 373: “Reduction in estimation error” is a bit misleading. It sounds like participants became more accurate after tool-use training, when they were really underestimating differences. Please reword here and throughout the manuscript for clarity.

7. Lines 380-389: It seems the comparisons of TDJ estimation errors at post-test between different amounts of tool-use practice were evaluated with individual paired t-tests for each distance. Were corrections for number of t-tests performed applied? Why not opt for a more robust repeated measures ANOVA?

8. Lines 386-389: The authors claim that the “findings suggest that tool-use induced changes in tactile distance perception scale with the amount of sensorimtotor experience, consistent with the gradual recalibration of body representation through extended tool-use”, but I’m not convinced this conclusion can be drawn definitively from the data. The recalibrations occurred at the shortest (30) and longest (120) trial blocks that happened to be on the same day, while the 60-trial block on day 2 did not lead to any pre- to post-testing differences. From this point of view, it seems that there could be an initial recalibration in body representation following novel tool-use that becomes resistant to change with further tool-use, which is why effects are not seen consistently on day 2 of testing. If the 60+90 trials and 30+120 trials had been counterbalanced so they were split across day 1 and day 2, I would find the authors claim more convincing. Including either day of testing (day 1, day 2) or trial block order (1,2,3,4) as fixed effects in the linear model could help strengthen the authors’ claim.

9. Figures 5 & 7: Indicate significant differences with asterisks.

10. Lines 402-409: I can see that the line in Fig 6. Is very flat so I believe that there was a “consistent tendency to underestimate reaching distance across all tool-use trial blocks at baseline”. It would be good to prove this statistically with an ANOVA for the sake of rigor.

11. Lines 415-417: The methods section mentions that arm length was included as a covariate but is not reported in the model. Please clarify.

12. Lines 428-436: Comment 7 above also applies to this analysis. Isn’t an ANOVA with follow-up t-tests is more appropriate? At the least, corrections for the number of t-tests performed should be applied.

13. Lines 435-436: “After 120 trials, a decrease increase was observed”. Please fix.

14. Line 437-438: Please state the statistical test performed at the beginning of this paragraph for clarity (e.g., We performed a 3-way mixed ANOVA ….). It’s confusing to include both the analysis of pre- and post-test RDE error across the tool-use trial lengths and this ANOVA. Please justify including both or just report the ANOVA since it seems like the consistent changes in RDE error from pre- to post-test across tool-use trials can be explained by differences in arm length, which is the main conclusion drawn from the results.

Discussion

15. The authors claim that the TDJ results indicate “gradual recalibration of perceived forearm tactile distance” based on the greatest change in errors from pre- to post-testing occurring at 120 trials is quite strong since the recalibrations only consistently happened on day 1 of testing. Once participants returned on day 2 after having had extended experience with the tool, the recalibration was less reliable. The authors provide a potential explanation based on representational competition (lines 493-496), but alternatively couldn’t day of testing be driving the effects (see comment 8)? If participants’ body representations become resilient to tool-use training, this is a key insight that needs to be followed up with dedicated studies. That the same configuration of trials occurred on each day across participants (30 + 120 trials always on day 1, and 60 + 90 trials always on day 2) should be mentioned in the limitations section.

Reviewer #2: In the present study, Najafabadi & Kayser investigate whether tool-induced changes in body representation are dependent upon the amount of exposure to the tool the user had. They used the TDJ and RDE to measure perception and action-oriented representations, respectively. These tasks were measured before and after performing a VR-based tool use task with four different lengths (30, 60, 90, and 120 trials). Tool use changed responses to the TDJ and RDE, consistent with plasticity in body representation. The TDJ effects were dependent upon how long the tool was used, whereas the RDE effects were not. This pattern of results is taken as evidence for practice-dependent effects in perceptual body representation but not action-oriented representation.

The present study is interesting and addresses an often-ignored topic: How much tool use is needed to change body representation? The results would therefore be interesting to those in the field. However, there are several shortcomings with the study in its current form that prevent me from currently endorsing it for publication. I list these below.

Major points

1) Throughout the paper, a change in somatosensory perception (here, tactile distance) is taken as evidence for a shrinkage of the represented limb size. This interpretation is controversial and should be discussed as such. Whereas some have interpreted the change as a shrinkage (e.g., Miller et al., 2014), many interpret the change as an increase in represented size (e.g., Cardinali, Martel, Sposito, Garbarini, etc.). I do not personally think there is a definitive answer to this question, especially since it seems to depend on the paradigm being used (e.g, tactile localization, midpoint bisection, TDJ). I suggest the authors be a bit more cautious in their interpretation, especially when discussing prior research.

2) It is not immediately clear to me how patterns of error relate to distortions of plasticity in spatial representation (e.g., arm length, pps). Indeed, I had to really think through what the direction of an error meant in terms of the numerical judgments. A more straightforward characterization of the behavior, in my opinion, is to use a regression approach to directly analyze the magnitude responses of each stimulus conditions (e.g., as Green 1982 did for the TDJ). Such an analysis would allow one to extract the bias in responses as well as the slope, which I would interpret as a gain that links perception (i.e., in tactile distance) with the underlying metric representation. As the current paper is now, a change in the error after tool use could be either due to a change in the spatial representation or a general change in response bias. I suggest that the authors also include this complementary approach, as it would provide a fuller picture of the data.

3) I am not convinced that the current results, at least as presented, provide strong evidence for the presence of practice effects (in TDJ ) or their absence (in RDE). First, the authors assume that practice effects require a substantial number of trials (minimum 30) to accumulate towards a ceiling; hence their interpretation of the RDE task results. However, there is evidence that changes in PPS can happen almost immediately (see Holmes et al 2004). The authors cannot rule out practice effects in the RDE task that occur earlier than 30 trials. Second, from what I can tell, the different trial combinations (30+120, 60+90) were not counterbalanced across days. The baselines for the conditions on day 2 (60+90) look quite different than for day 1; can the authors rule out carry-over effects from the first day? Effects of motor learning can last several days, especially when the same task is involved. Third and relatedly, are the tool-use effects in the 60 and 90 trial conditions significantly different? Just eye-balling Figure 5 they look quite similar. Finally, are there carryover effects between the two conditions of the same day? There is EEG evidence that effects of tool use on somatosensory brain responses can last at least 30 minutes after tool use (e.g., Miller et al. 2019 JoCN). Any effect from one treatment condition may therefore carryover to the baseline of the other condition, making interpretation difficult.

4) The VR results of the current study have implications for the types of sensory or sensorimotor information that drives changes to body representation, which has been ongoing discussion in the literature. Whereas some have argued for an important role for visual feedback (e.g., visual illusions in Miller et al., 2017 and Guerraz et al., 2018) others have stressed the importance of somatosensory feedback (e.g., Martel et al., 2019; Miller et al., 2018, Bahmad et al., 2020). Readers would benefit from an in-depth discussion of this, as the current study has important implications for it.

Minor points

5) The way PPS is discussed feels a bit inconsistent to me. In section 1.1., the RDE is said to be a proxy for measuring PPS and also a global action-oriented representation of the body, which to me sounds a bit like body schema (and not PPS). Can the authors clarify what they mean by PPS and what RDE is thought to measure.

6) I don’t follow the reasoning in the final sentence of the abstract, specifically this portion: “…the perceived metric of the limb itself appears to require cumulative sensorimotor evidence to overcome stable biological priors.” Significant effects were found at 30 trials. Though the magnitude of the results was cumulative, the term “overcome” suggests to me that there is a clear temporal point where the results go from no change to significant change. Please clarify this, in the abstract but also throughout the text where necessary.

**********

what does this mean?). If published, this will include your full peer review and any attached files.

If you choose “no”, your identity will remain anonymous but your review may still be made public.

**Do you want your identity to be public for this peer review?**  For information about this choice, including consent withdrawal, please see our Privacy Policy

Reviewer #1: No

Reviewer #2: No

**********

[NOTE: If reviewer comments were submitted as an attachment file, they will be attached to this email and accessible via the submission site. Please log into your account, locate the manuscript record, and check for the action link "View Attachments". If this link does not appear, there are no attachment files.]

To ensure your figures meet our technical requirements, please review our figure guidelines: https://journals.plos.org/plosone/s/figures

You may also use PLOS’s free figure tool, NAAS, to help you prepare publication quality figures: https://journals.plos.org/plosone/s/figures#loc-tools-for-figure-preparation.

NAAS will assess whether your figures meet our technical requirements by comparing each figure against our figure specifications.

# Author comment: Author response to Decision Letter 1

**DOI:** 10.1371/journal.pone.0361297.r002

11 Aug 2026

Please see the cover letter and attached response letter to reviewers for point-by-point revisions.

Attachment Submitted filename: Response Letter_Submission.docx

# Peer review: Decision Letter 1

**DOI:** 10.1371/journal.pone.0361297.r003

**Authors:** Patrick Bruns

8 Sep 2026

Dear Dr. Jahanian Najafabadi,

Thank you for submitting your manuscript to PLOS One. After careful consideration, we feel that it has merit but does not fully meet PLOS One’s publication criteria as it currently stands. Therefore, we invite you to submit a revised version of the manuscript that addresses the points raised during the review process.

As you will see below, while both reviewers appreciated the revisions, Reviewer #1 has a few remaining minor concerns that should be addressed.

Please submit your revised manuscript by Oct 23 2026 11:59PM. If you will need more time than this to complete your revisions, please reply to this message or contact the journal office at plosone@plos.org. When you're ready to submit your revision, log on to https://www.editorialmanager.com/pone/ and select the 'Submissions Needing Revision' folder to locate your manuscript file.

- A letter that responds to each point raised by the academic editor and reviewer(s). You should upload this letter as a separate file labeled 'Response to Reviewers'.
- A marked-up copy of your manuscript that highlights changes made to the original version. You should upload this as a separate file labeled 'Revised Manuscript with Track Changes'.
- An unmarked version of your revised paper without tracked changes. You should upload this as a separate file labeled 'Manuscript'.

If you would like to make changes to your financial disclosure, please include your updated statement in your cover letter. Guidelines for resubmitting your figure files are available below the reviewer comments at the end of this letter.

If applicable, we recommend that you deposit your laboratory protocols in protocols.io to enhance the reproducibility of your results. Protocols.io assigns your protocol its own identifier (DOI) so that it can be cited independently in the future. For instructions see: https://journals.plos.org/plosone/s/submission-guidelines#loc-laboratory-protocols. Additionally, PLOS One offers an option for publishing peer-reviewed Lab Protocol articles, which describe protocols hosted on protocols.io. Read more information on sharing protocols at https://plos.org/protocols?utm_medium=editorial-email&utm_source=authorletters&utm_campaign=protocols.

As the corresponding author, your ORCID iD is verified in the submission system and will appear in the published article. PLOS supports the use of ORCID, and we encourage all coauthors to register for an ORCID iD and use it as well. Please encourage your coauthors to verify their ORCID iD within the submission system before final acceptance, as unverified ORCID iDs will not appear in the published article. *Only*  the individual author can complete the verification step; PLOS staff *cannot*  verify ORCID iDs on behalf of authors.

We look forward to receiving your revised manuscript.

Kind regards,

Patrick Bruns

Academic Editor

PLOS One

Journal Requirements:

If the reviewer comments include a recommendation to cite specific previously published works, please review and evaluate these publications to determine whether they are relevant and should be cited. There is no requirement to cite these works unless the editor has indicated otherwise.

Please review your reference list to ensure that it is complete and correct. If you have cited papers that have been retracted, please include the rationale for doing so in the manuscript text, or remove these references and replace them with relevant current references. Any changes to the reference list should be mentioned in the rebuttal letter that accompanies your revised manuscript. If you need to cite a retracted article, indicate the article’s retracted status in the References list and also include a citation and full reference for the retraction notice.

[Note: HTML markup is below. Please do not edit.]

Reviewers' comments:

Reviewer's Responses to Questions

**Comments to the Author**

Reviewer #1: (No Response)

Reviewer #2: All comments have been addressed

**********

2. Is the manuscript technically sound, and do the data support the conclusions?

Reviewer #1: Yes

Reviewer #2: (No Response)

**********

3. Has the statistical analysis been performed appropriately and rigorously?

Reviewer #1: Yes

Reviewer #2: (No Response)

**********

4. Have the authors made all data underlying the findings in their manuscript fully available?

The PLOS Data policy

Reviewer #1: No

Reviewer #2: (No Response)

**********

5. Is the manuscript presented in an intelligible fashion and written in standard English?

Reviewer #1: Yes

Reviewer #2: (No Response)

**********

Reviewer #1: A wholehearted thank you to the authors for carefully considering each of my comments. I know it's painstaking work and I appreciate your open-mindedness and rigor. I have two outstanding points to address before I fully endorse the manuscript for publication.

First, PLOS One's policy is that the data be made fully available without restriction before publication, but the data availability statement reads that the data will be available on OSF.org only upon publication. Could the authors please share their data ahead of publication to conform to the journal's open science policies? See below for my remaining 2 concerns:

3. Lines 204-206: Were day 1 and day 2 consecutive? Was the delay between the two sessions the same for all participants?

Response: The testing days were non-consecutive and separated by a gap of minimum one week based on participants’ availability. We have updated the text to explicitly state this, which served as a reliable behavioral washout period between testing weeks.

The following has been added to the manuscript (lines 249-269):

“To isolate the effects of training duration and minimize carry-over between blocks, a 30minute rest period was imposed between the two practice blocks on the same day, in line with recent tool-use research (Jahanian Najafabadi et al., 2023b, 2023a; Jahanian Najafabadi, Rastegari, Imani, et al., 2026; Jahanian Najafabadi, Rastegari, & Longo, 2026; Jahanian Najafabadi & Godde, 2025). This washout interval was selected based on previous evidence indicating that tool-use–induced modulations of somatosensory cortical responses can persist for approximately 10 minutes following training (Miller et al., 2019a). In addition, the order of the two tool-use training blocks (e.g. 30 and 120) within each testing day was counterbalanced across participants to control for potential order effects. On Day 1, the blocks consisted of 30 and 120 trials; on Day 2, of 60 and 90 training trials.

To further reduce carry-over effects between testing days, the two days were separated by at least one week. Previous research has shown that somatosensory perceptual changes induced by brief periods of motor learning can remain detectable for at least 24 hours (Ostry et al., 2010). Although the persistence of virtual tool-use effects was not directly examined in the present study, this evidence suggests that training-induced sensorimotor adaptations may extend beyond the immediate post-training interval. The one-week separation was therefore implemented to minimize residual effects of prior training and increase the independence of the two testing days. Taken together, the washout interval, within each testing day counterbalancing (between two-blocks), and multi-day separation were implemented to minimize contamination between training conditions and ensure that observed changes could be attributed primarily to the experimental manipulations rather than residual effects of previous tool-use experience.”

Reviewer Response: Thank you for adding the above details to the methods section. Can the authors be more specific than “at least one week”? What was the maximum number of days between sessions? What was the mean and standard deviation? If there is a lot of variation between participants, couldn’t this have impacted the results? Longer washout, less carry over effects?

5. Lines 352-358: The 30 and 120 trial blocks were always on day one and 60 and 90 trial blocks were always on day 2. So, the 30/120 meant that participants always had less experience with the tool compared to 60 /90. Did the authors consider adding a fixed effect for testing day (1 or 2) or training block order (1-4). This could be a good idea especially since the fixed effects only accounted for a small proportion of the variance in the model.

Response: Thank you for your comment. We agree that a fully crossed design, in which all trial blocks were distributed across both testing days, would provide the strongest control for potential day-related influences. In the present study, however, trial blocks were counterbalanced within testing days rather than across days. Importantly, several observations suggest that the reported practice-dependent effects cannot be readily explained by a day confound. First, baseline analyses revealed no Trial Block × Distance interaction, suggesting that the pattern of tactile distance distortion was not very different across blocks. Second, if testing day were the primary driver of the observed effects, similar outcomes would be expected for the two blocks administered on the same day. This was not observed. Specifically, the 60trial condition did not show a significant pre-post change, whereas the 90-trial condition, which was administered within the same testing session, did show a significant change. Likewise, the strongest effect emerged in the 120-trial condition. These findings suggest that the observed differences cannot be attributed solely to day-related variability and are more consistent with a practice-dependent influence of tool-use training on tactile distance perception.

We nevertheless acknowledge that the partial confounding of trial block and testing day represents a limitation of the design and have added this point to the Discussion lines 751-754:

“Although testing sessions were separated by at least one week and block order was counterbalanced within each session, trial blocks were not fully crossed across testing days. Consequently, we cannot completely exclude the possibility that day-related variability contributed to some of the observed block differences.”

Reviewer Response: Thank you for explaining your logic and including my comment in the discussion. I find the reasoning persuasive but would find the results of linear mixed model with either a fixed effect for testing day (1 or 2) or training block order (1-4) even more convincing. If the results are unchanged, this point wouldn’t even need to be mentioned as a limitation in the discussion. It would strengthen the results.

Minor comment:

p. 19, line 594: “entered to the model as covariate” � “entered into the model as a covariate”

Reviewer #2: (No Response)

**********

what does this mean?). If published, this will include your full peer review and any attached files.

If you choose “no”, your identity will remain anonymous but your review may still be made public.

**Do you want your identity to be public for this peer review?**  For information about this choice, including consent withdrawal, please see our Privacy Policy

Reviewer #1: No

Reviewer #2: No

**********

[NOTE: If reviewer comments were submitted as an attachment file, they will be attached to this email and accessible via the submission site. Please log into your account, locate the manuscript record, and check for the action link "View Attachments". If this link does not appear, there are no attachment files.]

To ensure your figures meet our technical requirements, please review our figure guidelines: https://journals.plos.org/plosone/s/figures

You may also use PLOS’s free figure tool, NAAS, to help you prepare publication quality figures: https://journals.plos.org/plosone/s/figures#loc-tools-for-figure-preparation.

NAAS will assess whether your figures meet our technical requirements by comparing each figure against our figure specifications.

# Author comment: Author response to Decision Letter 2

**DOI:** 10.1371/journal.pone.0361297.r004

13 Sep 2026

Response Letter

Reviewers' comments:

Reviewer's Responses to Questions

Comments to the Author

4. Have the authors made all data underlying the findings in their manuscript fully available?

The PLOS Data policy requires authors to make all data underlying the findings described in their manuscript fully available without restriction, with rare exception (please refer to the Data Availability Statement in the manuscript PDF file). The data should be provided as part of the manuscript or its supporting information, or deposited to a public repository. For example, in addition to summary statistics, the data points behind means, medians and variance measures should be available. If there are restrictions on publicly sharing data—e.g. participant privacy or use of data from a third party—those must be specified.

Reviewer #1: No

Reviewer #2: (No Response)

6. Review Comments to the Author

Please use the space provided to explain your answers to the questions above. You may also include additional comments for the author, including concerns about dual publication, research ethics, or publication ethics. (Please upload your review as an attachment if it exceeds 20,000 characters)

Reviewer #1: A wholehearted thank you to the authors for carefully considering each of my comments. I know it's painstaking work and I appreciate your open-mindedness and rigor. I have two outstanding points to address before I fully endorse the manuscript for publication.

Response: We highly appreciated your consideration and feedback improving our work.

First, PLOS One's policy is that the data be made fully available without restriction before publication, but the data availability statement reads that the data will be available on OSF.org only upon publication. Could the authors please share their data ahead of publication to conform to the journal's open science policies? See below for my remaining 2 concerns:

Response: Thank you for your comment. We have now uploaded datasets through OSF and revised the text accordingly.

3. Lines 204-206: Were day 1 and day 2 consecutive? Was the delay between the two sessions the same for all participants?

Response: The testing days were non-consecutive and separated by a gap of minimum one week based on participants’ availability. We have updated the text to explicitly state this, which served as a reliable behavioral washout period between testing weeks.

The following has been added to the manuscript (lines 249-269):

“To isolate the effects of training duration and minimize carry-over between blocks, a 30minute rest period was imposed between the two practice blocks on the same day, in line with recent tool-use research (Jahanian Najafabadi et al., 2023b, 2023a; Jahanian Najafabadi, Rastegari, Imani, et al., 2026; Jahanian Najafabadi, Rastegari, & Longo, 2026; Jahanian Najafabadi & Godde, 2025). This washout interval was selected based on previous evidence indicating that tool-use–induced modulations of somatosensory cortical responses can persist for approximately 10 minutes following training (Miller et al., 2019a). In addition, the order of the two tool-use training blocks (e.g. 30 and 120) within each testing day was counterbalanced across participants to control for potential order effects. On Day 1, the blocks consisted of 30 and 120 trials; on Day 2, of 60 and 90 training trials.

To further reduce carry-over effects between testing days, the two days were separated by at least one week. Previous research has shown that somatosensory perceptual changes induced by brief periods of motor learning can remain detectable for at least 24 hours (Ostry et al., 2010). Although the persistence of virtual tool-use effects was not directly examined in the present study, this evidence suggests that training-induced sensorimotor adaptations may extend beyond the immediate post-training interval. The one-week separation was therefore implemented to minimize residual effects of prior training and increase the independence of the two testing days. Taken together, the washout interval, within each testing day counterbalancing (between two-blocks), and multi-day separation were implemented to minimize contamination between training conditions and ensure that observed changes could be attributed primarily to the experimental manipulations rather than residual effects of previous tool-use experience.”

Reviewer Response: Thank you for adding the above details to the methods section. Can the authors be more specific than “at least one week”? What was the maximum number of days between sessions? What was the mean and standard deviation? If there is a lot of variation between participants, couldn’t this have impacted the results? Longer washout, less carry over effects?

Response: Thank you for your comment. As requested, we clarified and better phrased the exact detail in the respected section. The one-week period was ±1 day to make sure participants find a time to participate considering their student classes and their assignments.

5. Lines 352-358: The 30 and 120 trial blocks were always on day one and 60 and 90 trial blocks were always on day 2. So, the 30/120 meant that participants always had less experience with the tool compared to 60 /90. Did the authors consider adding a fixed effect for testing day (1 or 2) or training block order (1-4). This could be a good idea especially since the fixed effects only accounted for a small proportion of the variance in the model.

Response: Thank you for your comment. We agree that a fully crossed design, in which all trial blocks were distributed across both testing days, would provide the strongest control for potential day-related influences. In the present study, however, trial blocks were counterbalanced within testing days rather than across days. Importantly, several observations suggest that the reported practice-dependent effects cannot be readily explained by a day confound. First, baseline analyses revealed no Trial Block × Distance interaction, suggesting that the pattern of tactile distance distortion was not very different across blocks. Second, if testing day were the primary driver of the observed effects, similar outcomes would be expected for the two blocks administered on the same day. This was not observed. Specifically, the 60trial condition did not show a significant pre-post change, whereas the 90-trial condition, which was administered within the same testing session, did show a significant change. Likewise, the strongest effect emerged in the 120-trial condition. These findings suggest that the observed differences cannot be attributed solely to day-related variability and are more consistent with a practice-dependent influence of tool-use training on tactile distance perception.

We nevertheless acknowledge that the partial confounding of trial block and testing day represents a limitation of the design and have added this point to the Discussion lines 751-754:

“Although testing sessions were separated by at least one week and block order was counterbalanced within each session, trial blocks were not fully crossed across testing days. Consequently, we cannot completely exclude the possibility that day-related variability contributed to some of the observed block differences.”

Reviewer Response: Thank you for explaining your logic and including my comment in the discussion. I find the reasoning persuasive but would find the results of linear mixed model with either a fixed effect for testing day (1 or 2) or training block order (1-4) even more convincing. If the results are unchanged, this point wouldn’t even need to be mentioned as a limitation in the discussion. It would strengthen the results.

Response: Thank you for your comment. You are correct, we have now removed the related statement from limitation section.

Minor comment: p. 19, line 594: “entered to the model as covariate” � “entered into the model as a covariate”

„a“ has been added.

Attachment Submitted filename: Response to Reviewers.docx

# Peer review: Decision Letter 2

**DOI:** 10.1371/journal.pone.0361297.r005

**Authors:** Patrick Bruns

8 Oct 2026

Practice-dependent changes in body representation but not in reaching distance following virtual tool-use

PONE-D-26-14462R2

Dear Dr. Jahanian Najafabadi,

We’re pleased to inform you that your manuscript has been judged scientifically suitable for publication and will be formally accepted for publication once it meets all outstanding technical requirements.

Within one week, you’ll receive an e-mail detailing the required amendments. When these have been addressed, you’ll receive a formal acceptance letter and your manuscript will be scheduled for publication.

An invoice will be generated when your article is formally accepted. Please note, if your institution has a publishing partnership with PLOS and your article meets the relevant criteria, all or part of your publication costs will be covered. Please make sure your user information is up-to-date by logging into Editorial Manager at Editorial Manager® and clicking the ‘Update My Information' link at the top of the page. For questions related to billing, please contact billing support.

If your institution or institutions have a press office, please notify them about your upcoming paper to help maximize its impact. If they’ll be preparing press materials, please inform our press team as soon as possible -- no later than 48 hours after receiving the formal acceptance. Your manuscript will remain under strict press embargo until 2 pm Eastern Time on the date of publication. For more information, please contact onepress@plos.org.

Kind regards,

Patrick Bruns

Academic Editor

PLOS One

Additional Editor Comments (optional):

Reviewers' comments:

Reviewer's Responses to Questions

**Comments to the Author**

Reviewer #1: All comments have been addressed

**********

2. Is the manuscript technically sound, and do the data support the conclusions?

Reviewer #1: (No Response)

**********

3. Has the statistical analysis been performed appropriately and rigorously?

Reviewer #1: (No Response)

**********

4. Have the authors made all data underlying the findings in their manuscript fully available?

The PLOS Data policy

Reviewer #1: (No Response)

**********

5. Is the manuscript presented in an intelligible fashion and written in standard English?

Reviewer #1: (No Response)

**********

Reviewer #1: (No Response)

**********

what does this mean?). If published, this will include your full peer review and any attached files.

If you choose “no”, your identity will remain anonymous but your review may still be made public.

**Do you want your identity to be public for this peer review?**  For information about this choice, including consent withdrawal, please see our Privacy Policy

Reviewer #1: No

**********

# Editor report: Acceptance letter

**DOI:** 10.1371/journal.pone.0361297.r006

**Authors:** Patrick Bruns

PONE-D-26-14462R2

PLOS One

Dear Dr. Jahanian Najafabadi,

I'm pleased to inform you that your manuscript has been deemed suitable for publication in PLOS One. Congratulations! Your manuscript is now being handed over to our production team.

At this stage, our production department will prepare your paper for publication. This includes ensuring the following:

* All references, tables, and figures are properly cited

* All relevant supporting information is included in the manuscript submission,

* There are no issues that prevent the paper from being properly typeset

You will receive further instructions from the production team, including instructions on how to review your proof when it is ready. Please keep in mind that we are working through a large volume of accepted articles, so please give us a few days to review your paper and let you know the next and final steps.

Lastly, if your institution or institutions have a press office, please let them know about your upcoming paper now to help maximize its impact. If they'll be preparing press materials, please inform our press team within the next 48 hours. Your manuscript will remain under strict press embargo until 2 pm Eastern Time on the date of publication. For more information, please contact onepress@plos.org.

You will receive an invoice from PLOS for your publication fee after your manuscript has reached the completed accept phase. If you receive an email requesting payment before acceptance or for any other service, this may be a phishing scheme. Learn how to identify phishing emails and protect your accounts at https://explore.plos.org/phishing.

If we can help with anything else, please email us at customercare@plos.org.

Thank you for submitting your work to PLOS One and supporting open access.

Kind regards,

PLOS One Editorial Office Staff

on behalf of

Dr. Patrick Bruns

Academic Editor

PLOS One
